# Visualizing YouTube Commenters’ Conceptions of the US Health Care System: Semantic Network Analysis Method for Evidence-Based Policy Making

**DOI:** 10.2196/58227

**Published:** 2025-02-11

**Authors:** Lana V Ivanitskaya, Elina Erzikova

**Affiliations:** 1 Health Administration, School of Health Sciences The Herbert H. and Grace A. Dow College of Health Professions Central Michigan University Mount Pleasant, MI United States; 2 Strategic Communication, School of Communication, Journalism and Media College of the Arts and Media Central Michigan University Mount Pleasant, MI United States

**Keywords:** social media, semantic network, health system, health policy, ideology, VOSviewer, health care reform, health services, health care workforce, health insurance

## Abstract

**Background:**

The challenge of extracting meaningful patterns from the overwhelming noise of social media to guide decision-makers remains largely unresolved.

**Objective:**

This study aimed to evaluate the application of a semantic network method for creating an interactive visualization of social media discourse surrounding the US health care system.

**Methods:**

Building upon bibliometric approaches to conducting health studies, we repurposed the VOSviewer software program to analyze 179,193 YouTube comments about the US health care system. Using the overlay-enhanced semantic network method, we mapped the contents and structure of the commentary evoked by 53 YouTube videos uploaded in 2014 to 2023 by right-wing, left-wing, and centrist media outlets. The videos included newscasts, full-length documentaries, political satire, and stand-up comedy. We analyzed term co-occurrence network clusters, contextualized with custom-built information layers called overlays, and performed tests of the semantic network’s robustness, representativeness, structural relevance, semantic accuracy, and usefulness for decision support. We examined how the comments mentioning 4 health system design concepts—universal health care, Medicare for All, single payer, and socialized medicine—were distributed across the network terms.

**Results:**

Grounded in the textual data, the macrolevel network representation unveiled complex discussions about illness and wellness; health services; ideology and society; the politics of health care agendas and reforms, market regulation, and health insurance; the health care workforce; dental care; and wait times. We observed thematic alignment between the network terms, extracted from YouTube comments, and the videos that elicited these comments. Discussions about illness and wellness persisted across time, as well as international comparisons of costs of ambulances, specialist care, prescriptions, and appointment wait times. The international comparisons were linked to commentaries with a higher concentration of British-spelled words, underscoring the global nature of the US health care discussion, which attracted domestic and global YouTube commenters. Shortages of nurses, nurse burnout, and their contributing factors (eg, shift work, nurse-to-patient staffing ratios, and corporate greed) were covered in comments with many likes. Comments about universal health care had much higher use of ideological terms than comments about single-payer health systems.

**Conclusions:**

YouTube users addressed issues of societal and policy relevance: social determinants of health, concerns for populations considered vulnerable, health equity, racism, health care quality, and access to essential health services. Versatile and applicable to health policy studies, the method presented and evaluated in our study supports evidence-based decision-making and contextualized understanding of diverse viewpoints. Interactive visualizations can help to uncover large-scale patterns and guide strategic use of analytical resources to perform qualitative research.

## Introduction

### Background

The US health care system, characterized by high costs [1] and perceived to fall “far short of its potential” [2], has been a focal point for media attention and public commentary over the past decade. Discussions have revolved around topics such as the repeal of Obamacare, presidential health care agendas, the exorbitant costs of health care, comparisons to systems in other nations, and postpandemic health care personnel shortages. Throughout this period, conservative, moderate, and liberal media outlets have produced a variety of content, including newscasts, full-length documentaries, political satire, and stand-up comedy, all centered on the intricacies of the US health care system [3-6]. When disseminated through YouTube (Google Inc), the most popular platform among US social media users [7], select videos have generated millions of views and tens of thousands of comments. To the best of our knowledge, the perspectives of YouTube commenters on the US health care system and its reform, despite their considerable value for policy analysis, remain unexplored.

### Objectives

Social media discussions are abundant, but they are often chaotic, noisy, indignant, and hateful [8-11]. There is a need for a method that effectively visualizes large volumes of commentary, filters out the noise, and highlights key patterns, making the information more digestible for stakeholders. The current state of social media research falls short of efficiently and clearly disseminating scientific outputs to diverse audiences. In quantitative social media studies, the constraints are statistical and graphical outputs with low idea density or high decoding requirements, which often require specialized knowledge. In qualitative studies, researchers communicate analytical outputs as summaries of themes and subthemes with representative quotes; however, they are based on limited data samples.

To address these challenges, we propose a mixed methods approach of mapping social media commentary. This approach combines automation and human judgment to create a visual representation of social media comments’ contents and structure, presenting them as a semantic network [12]. This methodology is particularly relevant for researchers, policy makers, and the wider public seeking a better understanding of complex social media narratives. We repurpose VOSviewer (Centre for Science and Technology Studies at Leiden University), a user-friendly bibliometric tool, to analyze tens of thousands of social media comments on YouTube regarding the US health care system. In this study, semantic networks are graphical representations of social media comment meanings. Nodes represent terms frequently mentioned in YouTube comments, linked and grouped into clusters based on their co-occurrence.

Since their introduction in 2010, VOSviewer algorithms have been extensively applied to build term co-occurrence networks from the text of article titles and abstracts [13-20]. Visualization of nonbibliometric textual data as semantic networks in VOSviewer was proposed in 2011 [21], followed by early visualizations of Twitter and YouTube discussions ([22-25]). Subsequent explorations of VOSviewer’s applications to social media comments and hashtags primarily led to cluster mapping ([26-35]). Notably, some scholars enhanced their cluster maps with informational layers called custom overlays to reveal patterns not visible in the base network [36-38].

Previous research compared VOSviewer semantic networks to networks generated from manually coded Twitter text [26]. However, there have been few systematic evaluations of VOSviewer-generated semantic networks derived from social media data. Consequently, our overarching goal is to evaluate VOSviewer’s application to social media data: Can it produce credible semantic networks to be used as analytical and communication tools? We test VOSviewer’s term co-occurrence map with custom-built overlays by posing 3 research questions:

1. How well does the VOSviewer network capture the content, context, and structure of social media comments?
2. What does it reveal about a decade-long online public discussion of the US health care system?
3. What is the policy analysis value of VOSviewer visualizations?

## Methods

### Semantic Network Construction

VOSviewer generates a custom semantic network by processing a corpus text file featuring social media comments. Our corpus comprised the text of primary comments and first-level replies to 53 videos shared by 17 US-based media outlets on their respective YouTube platforms between 2014 and 2023. The videos were sourced from news outlets such as Consumer News and Business Channel, Cable News Network, Fox News, and Public Broadcasting Service Frontline. Detailed criteria for video selection and video characteristics are outlined in the Tables S1 and S2 in Multimedia Appendix 1 [39]. After eliminating 5575 duplicate comments from the initial dataset of primary comments at first-level responses, our final corpus encompassed a total of 179,193 unique comments.

VOSviewer processes YouTube comments by detecting sentences, applying the Apache Software Foundation’s OpenNLP library algorithm for part-of-speech tagging, identifying terms as nouns and the longest noun phrases, and unifying terms through various methods [17,18]. From an initial pool of 1948 terms appearing in at least 60 comments, a subset of 323 (16.58%) terms related to the US health care system, such as Obamacare, prescription, and wait time, was selected for the final semantic network. A detailed term selection process, including manual screening and thesaurus construction, is described in Multimedia Appendix 1.

By distilling 179,193 comments into a network with several hundred nodes, a macro model of YouTube video commentaries was created, providing insight into social media users’ discussions on US health care. In this network, terms are interconnected and organized into distinct, nonoverlapping clusters [15,19,20]. A cluster is a group of terms tightly linked within the group and loosely connected with terms outside it. If >1 term was extracted from the text of the comment, it is possible for the same comment to be represented by multiple nodes in multiple clusters. We did a thematic analysis of clusters to gain insights about the US health system discourse.

We addressed limitations observed in previously published semantic networks by enhancing the network’s informational value. First, we added custom overlays to VOSviewer’s map, which displays the color of network nodes based on selected attributes. To build overlays, we coded each comment to reflect the theme of its YouTube video and added these codes, along with other comment characteristics (eg, comment date), to a scores file, which was uploaded to the VOSviewer together with our corpus file that contained YouTube comments (for more information on building corpus and scores files, refer to Multimedia Appendix 1). Second, we presented findings with hyperlinks to VOSviewer Online for broader accessibility and interactive engagement with our semantic network.

### Network Interpretation and Evaluation

The evaluation of the US health care system’s semantic network and its overlays was structured as follows. A comparison of 2 networks, before and after the deletion of repeated comments, served as a test of network robustness. Thematic alignment between the network terms, extracted from YouTube comments, and the videos that elicited these comments was a test of network’s content representation.

To examine structural relevance, we asked if network relationships reflected the underlying meanings evident in YouTube comments. We examined clusters: Do terms in the same cluster have more similar meanings than terms in different clusters? We also examined pairs and groups of interconnected terms: Are they used together in the source data? Do their relationships align with existing knowledge? A comprehensive analysis of all pairs or term groups is outside of the scope of this study. For practical reasons, we engaged in close reading of a limited number of comments, focusing mainly on smaller nodes. When the number of comments exceeded 200, we randomly sampled 200 comments to cover discussions of different videos, taking care to sample more than once when we encountered heterogeneous ideas that required careful interpretation. When ≥2 nodes were examined, we used close reading of comments that mentioned all selected terms. Following the approach by Eve [40], network visualizations were used to locate “points of interest, which are then resynthesized into close readings.”

In addition, we performed tests of semantic accuracy through raw data verification. Specifically, we cross-checked ambiguous or unexpected terms in our network against the comments that mentioned them. The analysis involved multiple readings of each comment to capture nuances of how individuals articulate their experiences or opinions of the US health care system, focusing on the words that were extracted as terms, their meaning, and context. On several occasions, for example, when performing a close reading for ideology, we offered brief summaries of the main ideas expressed by the commenters. Our validation of semantic network findings against extant comments adhered to the principles for quantitative text analysis outlined by Grimmer and Stewart [41].

Finally, we tested the usefulness of semantic network analysis for generating policy-relevant insights. We picked 4 health system design concepts—universal health care, Medicare for All, single payer, and socialized medicine—and examined how the comments mentioning these concepts were distributed across the terms we mapped. For insights into the policy ramifications of public perceptions of health system design, we focused on ideological terms and those with the highest share of comments referring to each concept.

### Ethical Considerations

Ethics approval for this study was sought from Central Michigan University’s Institutional Review Board (project 2023-1021-Mt. P). The study did not meet the definition of human participant research under the purview of the institutional review board according to federal regulations. The study used publicly accessible user-generated YouTube comments. The data were deidentified and aggregated before analysis. As the results are presented in an aggregate form, individual commenters cannot be identified. Informed consent has not been obtained. No compensation was provided to comment contributors.

## Results

### A Semantic Network of Term Co-Occurrence and Clustering

**Figure 1:**
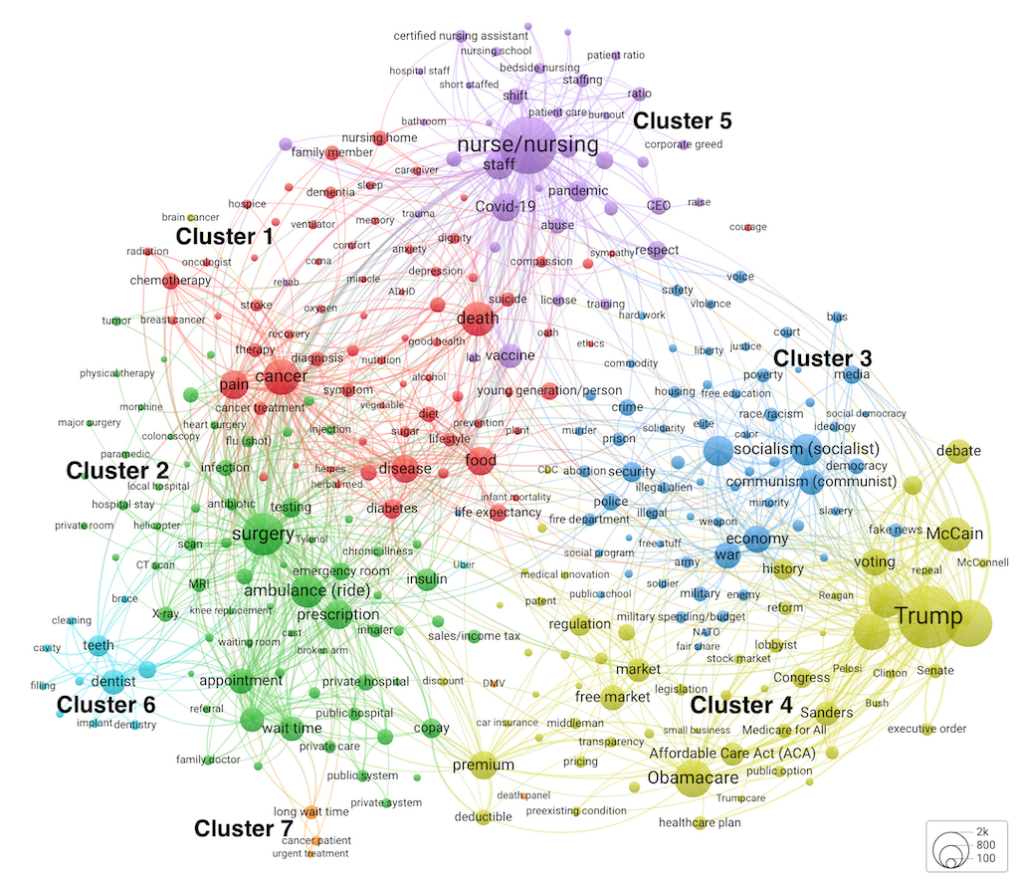
A co-occurrence network (cluster map) of terms extracted from the comments on 53 YouTube videos about the US health care system. Binary-counted terms that occurred ≥60 times were mapped. An interactive map is available from Leiden University’s VOSviewer app.

From a manually screened list of 539 terms occurring in our corpus at least 60 times, VOSviewer’s algorithm assisted in the selection of 323 (59.9%) most relevant terms [19]. Figure 1 [42] shows a 7-cluster solution for a term co-occurrence network.

On average, each term represented 357.74 (SD 606.88; median 163, IQR 104-321) comments. The longer the comment, the greater the likelihood that multiple terms were extracted from it. VOSviewer assigns cluster numbers based on the quantity of nodes; the same cluster numbers appear in our online interactive maps (URLs are provided in the notes of Figure 1).

Cluster 1 (red) emerged as the largest group of nodes, covering chronic diseases, treatment, pain, and death. Its diverse terms also included topics related to disease prevention (*diet, exercise,* and *smoking*), mental health (*ADHD* [attention-deficit/hyperactivity disorder,] *anxiety*, and *depression*), and end-of-life issues (*hospice, euthanasia,* and *do-not-resuscitate*). Below it, cluster 2 (green) terms covered services, encompassing surgeries, emergency medical services, procedures, diagnostics, wait times, and discussions about public versus private health organizations and prescription medications. On the right, cluster 3 (dark blue) had terms about political ideologies, economic, societal, and cultural issues, surrounded by nodes from cluster 4 (yellow) related to political actors, institutions, the 2010 Patient Protection and *Affordable Care Act* (*ACA* or *Obamacare*), market regulation, and insurance terminology. The top of the map displayed a group of terms (cluster 5, purple) dedicated to health worker shortages, nurse-to-patient ratios, and nurses’ burnout. Dental care terms formed a group on the lower left (cluster 6, light blue). Finally, a 5-node group (cluster 7, orange) at the bottom of the map had terms related to long wait times by patients with cancer who required urgent treatments, as well as further away terms *DMV* (Department of Motor Vehicles) and *death panel*.

The network displayed a rather coherent collection of terms, the meaning of which could be intuitively understood within the context of the US health care, with a few exceptions. For instance, as we manually selected terms for map inclusion, we checked the use of an ambiguous term *DMV* in YouTube users’ comments. *DMV* was mentioned as a metaphor in a debate of government-managed health care efficiency. It was retained due to its relevance to the health care discourse.

The interpretive value of our network extended beyond a simple list of terms. The network specified links between terms that were often mentioned together, for example, *pricing* and *transparency* in cluster 4. Meaning extraction was further aided by the analysis of spatial proximity, cluster assignment, and cluster boundaries. For example, *preexisting condition*, as a term of interest, was directly and most strongly linked to *Obamacare* and *ACA*, which were mentioned with *preexisting condition* in multiple comments*.* This finding was consistent with a key ACA provision: insurance companies cannot use applicants’ medical history to deny coverage or charge higher premiums based on their preexisting conditions [43]. Network structure’s alignment with existing knowledge speaks to its structural relevance. *Preexisting condition* is located close to *premium,* *deductible, pricing,* market-related terms, and *government regulation* from cluster 4 about politics, as well as to *private health insurance* and *copay* on the far right of cluster 2, which is mostly dedicated to health care services. Therefore, when YouTubers discussed the US health care system, they used a noun phrase *preexisting condition* at the semantic intersection of health care politics and legislation, insurance pricing, and health services access.

In summary, the 323 networked terms, identified as most relevant by VOSviewer, unveiled discussions about illness and wellness; health services; ideology and society; the politics of health care agendas and reforms, market regulation, and health insurance; health care workforce; dental care; and concerns such as long wait times.

Before we removed 5575 duplicate comments, our original cluster map (Figure S1 in Multimedia Appendix 1) was quite similar to the cluster map in Figure 1. Our inquiry into the medical debt cluster comments uncovered repeated comments by a single YouTube user. After deletion, this cluster disappeared, but the network’s overall structure largely remained intact, demonstrating its robustness.

Next, we examined clusters and nodes using overlays that reflected 2 aspects of the YouTube platform: the videos that elicited comments and the commentary itself. We assessed the usefulness of custom overlays as contextualization tools: Do they improve our understanding of nodes, node groups, and clusters? While we presented data on both video attributes and comment attributes, our analysis prioritized overlays depicting comment characteristics because they are more valuable for understanding digital publics’ discussion of the US health care system.

### Distribution of Video Groups Across Network Clusters

Thematic alignment between the video content that elicited the commentary and the commentary itself speaks to the content representativeness of the VOSviewer term co-occurrence network. The distribution of comments from 10 thematically diverse YouTube video groups across our term network is shown in overlays in Figure S2 in Multimedia Appendix 1. Our main findings are summarized in Table 1.

We observed substantial thematic congruence between video content and cluster terms. Nodes with above-average concentrations of comments related to the health care workforce were closely grouped in cluster 5, encompassing terms about nurses, staffing shortages, and management. Unlike most nodes in cluster 5, which were associated with health care workforce videos, the term *respect* had an above-average share of comments related to ACA and Obamacare reform videos. Our analysis of comments indicated that commenters mentioned respect for nurses, which explained the placement of *respect* in cluster 5. In addition, many comments on ACA and Obamacare reform videos expressed respect for Senator John McCain, which explained the connection between the term *respect* and *McCain*. *Respect*’s placement within cluster 5 but at its outer boundary, in the direction of node McCain, coupled with video overlay evidence, suggested semantic accuracy and structural relevance of our network.

Videos from 2 groups (health care policies, politics, ACA, and Obamacare reform) generated comments in cluster 4, which consisted of numerous political and reform-related terms. In addition, videos about health costs, one of which was titled “Dollars and Dentists,” elicited discussions of dental care (cluster 6). Comments on videos about health care systems in different countries produced terms that appeared in multiple clusters but mostly in cluster 2 about health services and cluster 7 about long wait time concerns. At the same time, a Home Box Office video “Medicare for All” featuring John Oliver and a Netflix video featuring stand-up comedians making jokes about the US health care produced comments in nodes scattered across the map. The Netflix video showcased many comedians and topics, one of whom, Wanda Sykes, spoke about opioids from the perspective of racial and ethnic minority people. A commentary on this topic appeared in nodes *pain* and *prescription* (left side of the map) and *race/racism, Black person*, and *White person* (right side of the map), where commenters debated racial disparities in pain medicine access. For race-related nodes, the share of comments on the Netflix video (comedy on the US health care) varied between 1% and 8%, indicating that it was not the only video prompting the discussion. This finding is not unique; it was common for terms to represent commentaries to a wide variety of videos or video groups.

Across all video group overlay legends, the highest scale midpoint was 0.25 for videos about health care costs and financial issues. It means that, on average, 25% (SD 14%) of comments within a term come from that video group. Across 323 map terms and 10 video theme overlays, there were only 11 (0.34%) instances (out of 3230 possible instances) where terms represented >90% of comments from a single video group.

**Table 1:** Characteristics of videos that elicited comments related to cluster-specific terms.

Cluster number (color)	Topical areas	Cluster’s 10 largest terms	Video groups that elicited comments related to most, some, or specific terms within a cluster
1 (red)	Illness and wellness, including mental health and end of life	*Cancer, death, pain, food, disease, diabetes, young generation/person, life expectancy, chemotherapy,* and *cure*	Children’s health care (some terms) End-of-life health care (some terms) Health care systems in different countries (*young generation/person* and *life expectancy*) Comedy on the US health care (*pain*) Medicare for All video by John Oliver (*pain*)
2 (green)	Health services	*Surgery, ambulance (ride), prescription, appointment, wait time, specialist, insulin, testing, copay,* and *emergency room*	Health care systems in different countries (most terms) Medicare for All video by John Oliver (most terms) Comedy on the US health care (*prescription*)
3 (dark blue)	Ideology and society	*Socialism (socialist), capitalism (capitalist), economy, war, communism (communist), security, media, police, crime,* and *democracy*	Single-payer health care (most terms) Health care systems in different countries (some terms) Medicare for All video by John Oliver (some terms) Health care costs and financial issues (*capitalism*) Comedy on the US health care (*race/racism, Black person,* and *White person*) ACA^a^/Obamacare reform (*race/racism, Black person,* and *White person*)
4 (yellow)	Health care politics, reform, market regulation, and insurance	*Trump, Biden, Obamacare, Republican, Democrat, McCain, premium, voting, free market,* and *debate*	Health care policies and politics (most terms) ACA/Obamacare reform (most terms) Medicare for All video by John Oliver (some terms) Single-payer health care (some terms) Health care costs and financial issues (market regulation terms)
5 (purple)	Health care workforce	*Nurse/nursing, staff, Covid-19, vaccine, pandemic, respect, shortage, management, CEO*^b^*,* and *shift*	Health care workforce (most terms) Health care systems in different countries (vaccine) ACA/Obamacare reform (*respect*)
6 (light blue)	Dental care	*Dentist, teeth, dental care, dentistry, implant, dental insurance, cleaning, cavity, filling,* and *brace*	Health care costs and financial issues (most terms)
7 (orange)	Concerns	*Long wait time, cancer patient, DMV*^c^*, urgent treatment,* and *death panel*	Health care systems in different countries (most terms) Single-payer health care (*DMV*)

^a^ACA: Affordable Care Act.

^b^CEO: chief executive officer.

^c^DMV: Department of Motor Vehicles.

### Comment Date and Ongoing Discussions

**Figure 2:**
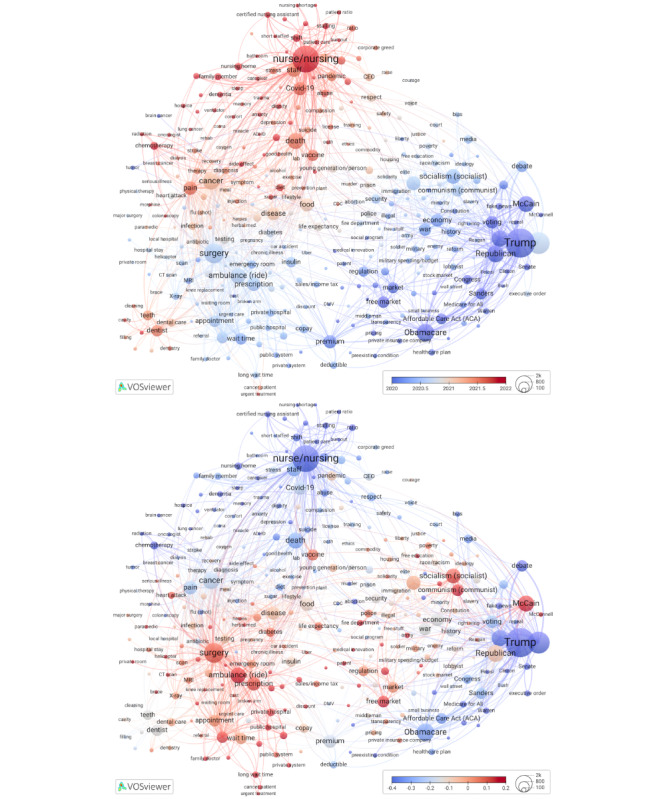
Overlays to Figure 1 for mean comment date (top) and ongoing discussions (standardized scores, bottom). High-resolution versions are available in Multimedia Appendix 1 (Figures S4 and S5).

When considering the timing of comments, the overall mean for all nodes was December 2020 (mean 2020.99, SD 0.81; range: from early 2018 for *repeal*, referring to the Trump administration and Republican lawmakers’ efforts to repeal the ACA, to early 2023 for *do-not-resuscitate*). Clusters 1, 5, and 6 have terms with more recent comments than other clusters (Figure 2, left [42]), which is likely a function of when a video was uploaded on YouTube.

Also shown in Figure 2 are ongoing discussions, conceptualized at the term level as mean posting time since the first comment in the respective video. We calculated time for each comment, based on the video it came from, then averaged across all comments behind each term. The terms that scored above the midpoint of 0.49 years (approximately 6 months) highlighted areas on the map where YouTube users continued to contribute comments long after the videos were posted, serving as a proxy for ongoing interest and engagement. Comment scores were calculated in 2 ways: without standardization, expressed as a fraction of a year (Figure S3 in Multimedia Appendix 1), and with standardization, using the base-10 logarithm to adjust for skewed data. The standardized scores were then normalized so that the mean is 0 and the scale points represent SDs (Figure 2, right).

Ongoing discussions in cluster 1, “illness and wellness,” were about cure (*herbal medicine* and *herpes*), *diabetes*, and life expectancy, and young people persisted, on average, for 11 months. In cluster 2, “health services,” ongoing discussions revolved around ambulances, specialist care, prescriptions, appointment wait times, copays, and private (vs public) health insurance or services, roughly covering the same area as high-scoring nodes in an overlay for videos about health care systems in different countries. YouTube commenters demonstrated continued interest in these topics. On average, cluster 2 terms that scored above the mean came from comments posted approximately 9 months after the first comment on a given video.

In cluster 3, “ideology and society,” YouTube users’ comments on political ideologies, police, and military were typically added around the 8-month mark, on average. To better understand an unexpectedly salient group of ideological terms in our map, we analyzed hundreds of comments about communism, socialism, and capitalism. Our analysis confirmed node size and interconnectedness. The discussion of the US health care system was highly politicized, with ideological battles that revolved around dichotomies, such as socialism versus capitalism. Individuals who self-identified as capitalist, conservative, libertarian, or Republican outright rejected any government involvement in health care, calling it socialism, which was often equated with communism (thus confirming node proximity), social democracy, inefficiency, economic decline, and excessive control. Commenters who self-identified as progressive, liberal, social democrat, or left leaning pointed out that health care in the United States was already a mix of capitalism and socialism: publicly funded US police and army were essentially socialized law enforcement, similar to socialized medicine in other countries. They saw no logical reason to reject socialized medicine.

Moreover, several non-US commenters and US residents living abroad shared their positive experiences with health systems in Europe and elsewhere, pointing out that they were affordable to residents with low-income status. Commenters questioned the following: Why do Americans accept *GoFundMe* fundraising to cover medical expenses but not universal health care? Those who defended capitalism praised it for *medical innovation* and high quality of health care but often added that it must be properly regulated. Application of capitalist principles to the US health care system was also discussed in connection to greed, lack of access to health care services, inequities, and poor outcomes. Multiple comments suggested that every economy needed a mix of socialism (relating it to public good or public welfare) and regulated capitalism to counterbalance corporate interests.

Finally, in cluster 4, “health care politics, reform, market regulation, and insurance,” we observed ongoing discussions about market-related topics (*monopoly, regulation,* and *market*) and especially the role of John McCain during Obamacare repeal.

### Comment Likes

Comment likes were standardized using the same method as for ongoing discussions. We examined overlays for cluster-specific concentrations of terms that scored above the mean, identified dyads of linked terms that scored high, and summarized the most-liked comments from a specific cluster or term.

**Figure 3:**
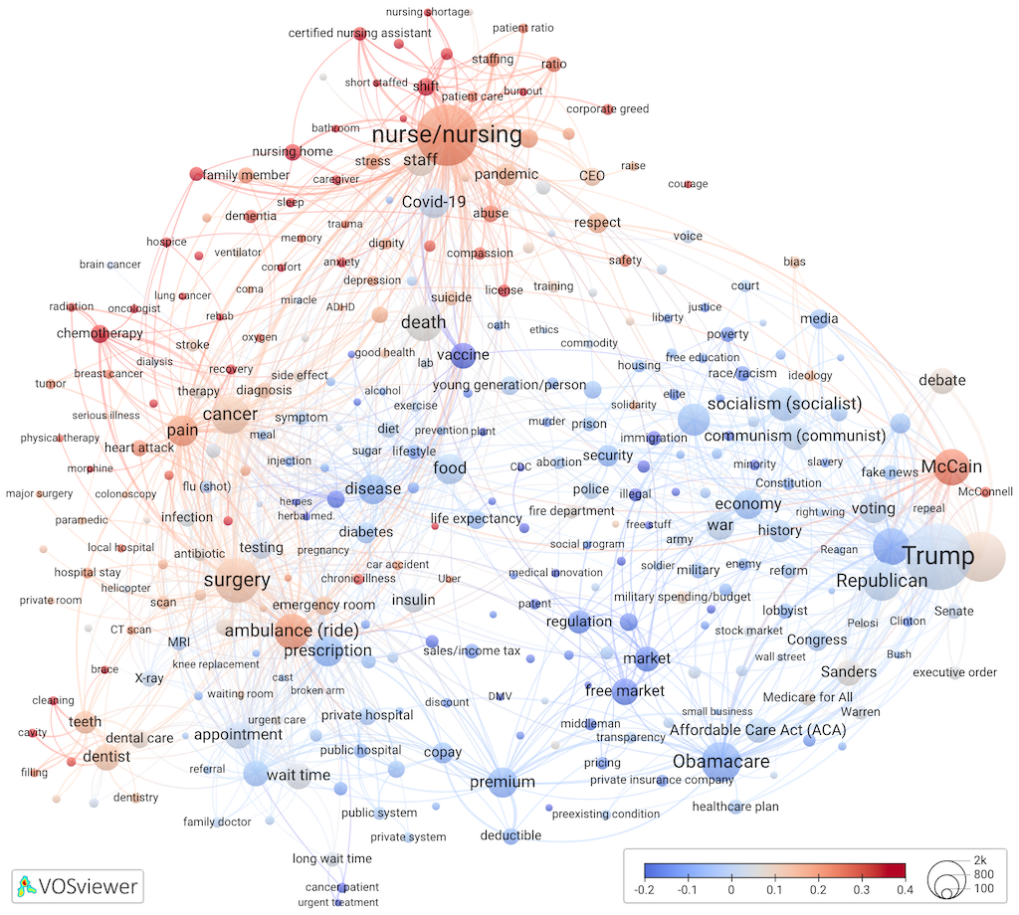
A mean comment likes (standardized) overlay to Figure 1.

In Figure 3 [42], the largest concentrations of above-average liked comments were mostly cluster specific (clusters 1, 2, 5, and 6). Most-liked cluster 5 terms came from comments about shortage of nurses and nurse burnout as well as factors contributing to it (*shift*, *short staffed, corporate greed, patient ratios, abuse,* and *management*). We checked an unexpected connection between *shift* (0.58 SD above the mean for all terms) and *bathroom* (0.48 SD above the mean), which represented highly liked comments. A total of 20 unique commenters shared stories of extreme job demands, describing how nurses worked long shifts, endured heavy workloads, faced high patient-to-nurse ratios, and had to wait for breaks to address their physiological needs. All but 3 commenters self-revealed their profession. They were experienced nurses, practicing or retired, or nursing students on clinical rotations. Their detail-rich comments described burnout antecedents, such as profits over staffing, mistreatment of nurses, and mandatory overtime, and outcomes, for example, reduced patient care quality and medication errors.

In cluster 1, cancer-related terms, the term *sleep*, and end-of-life terms such as *do-not-resuscitate* were extracted from comments with many likes. Individuals who mentioned “do not resuscitate” (DNR; 0.42) expressed deeply personal desires for autonomy and the avoidance of prolonged distress at the end of life. The commenters identified themselves as older adults, patient advocates, veterans, or health care workers. They discussed the implications of DNR orders, sometimes expressing doubts that an overburdened health care system could handle their implementation in a patient-centered way. Nevertheless, some nurses who witnessed slow deaths of patients without DNR orders chose to create their own advance directives.

Comments about *sleep* were also well liked (0.43) but, unlike the DNR discussion, referred to many different contexts: caregivers, including nurses, experiencing stressors and sleeplessness; sleep as a precondition to wellness; and in the context of passing away peacefully in one’s sleep. The placement of *sleep* within our network, on the boundary of cluster 1 terms (*dementia, family member, nursing home,* and *caregiver*) and cluster 5 terms (*stress, trauma*, and a direct link to *nurse/nursing)*, matched these observations and provided evidence of semantic accuracy and structural relevance.

Among dental treatment nodes in cluster 6, *cavity* scored the highest (0.48) on comments with likes. Cavity-related comments came from individuals who revealed the following self-identifications: residence (mostly the United States but also US residents living abroad and foreign nationals from multiple continents), low income (jobless or poor), and medical tourists (eg, US residents receiving dental treatments in Mexico). Commenters particularly liked quotes of low dental costs in Australia, France, Mexico, and other countries; stories of cost savings after buying airfare and paying for dental treatments abroad; personal accounts of dentists recommending unnecessary procedures; and oral health tips, such as reducing sugar intake. Comments specified systemic problems with US dental care: financial strains, even with dental insurance; potentially superfluous, according to second opinions, or unnecessarily extensive procedures (eg, on baby teeth); worsened conditions due to cost-related treatment delays; and processed sugar industry’s influence on consumption of foods, leading to dental decay.

Other clusters also had node groups that were well liked. We explored 2 dyads of linked nodes that scored high on likes: *McCain*–*McConnell* (0.31-0.34, cluster 4) and *ambulance (ride)–Uber* (0.26 for both, cluster 2), with above-average likes. In first dyad comments, most commenters applauded McCain’s vote that helped prevent the repeal of ACA and criticized McConnell and other Republicans. Comments from the second dyad, *ambulance* and *Uber,* were by YouTuber users who expressed concerns about the cost of US ambulances and Americans’ reluctance to use specialized emergency transportation. To avoid unpredictable costs, some US commenters planned to use nonmedical transport, such as ride-sharing services like Uber, during health emergencies.

### Comments With Select British Spellings

Figure S6 in Multimedia Appendix 1 displays an overlay that approximates contributions from commenters whose backgrounds are associated with regions where British spelling conventions are more common than in the United States. Such spelling was detected in multiple clusters, but the highest-scoring terms were in cluster 2 (*national insurance*, *government hospital*, and *private system*) and cluster 3 (*free education*, *unemployment,* and *justice*).

### Commonly Mentioned Health Care Concepts: System Design Ideas

**Figure 4:**
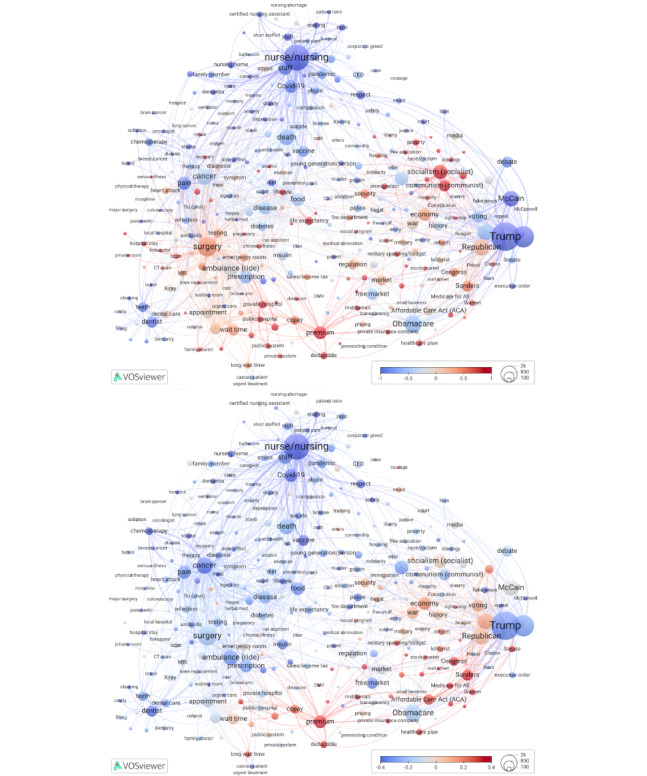
Overlays to Figure 1 depicting the distributions of comments that mention “universal health” (top) and “Medicare for All” (bottom). High-resolution versions are available in Multimedia Appendix 1 (Figures S7 and S8).

**Figure 5:**
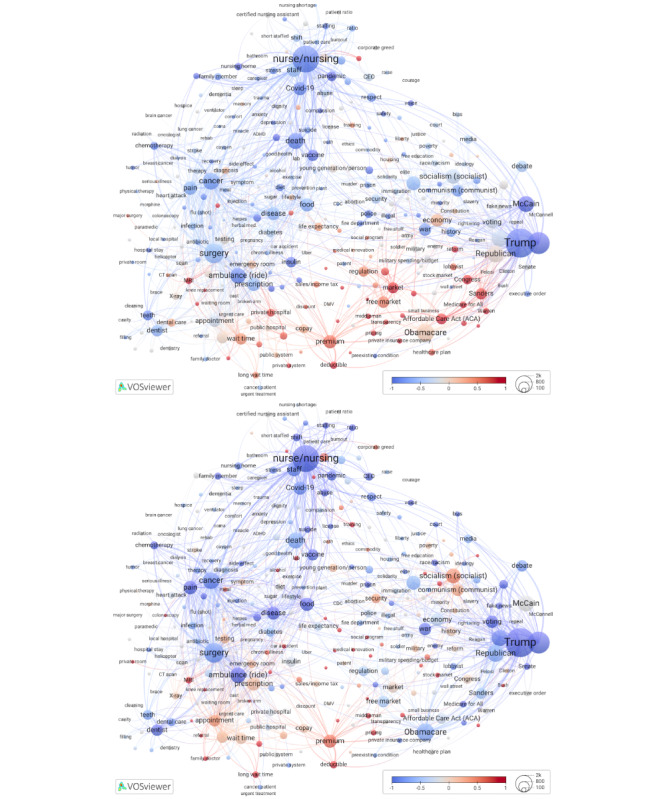
Overlays to Figure 1 depicting the distributions of comments that mention “single-payer” (top) and “socialized medicine” (bottom). High-resolution versions are available in Multimedia Appendix 1 (Figures S9 and S10).

Our last set of overlays demonstrates the distribution of comments that mention policy-relevant ideas on health care system design: universal health care, Medicare for All, a single-payer system, and socialized medicine (Table 2). VOSviewer Online offers a modifiable legend with an option to normalize term scores by subtracting mean and dividing by SD. When term scores are normalized, we can directly compare multiple overlays (Figures 4 and 5 [42]) to identify map areas with terms that are extracted from a high (vs low) share of comments mentioning specific system design ideas. Unlike the standardization of comment scores, normalization is performed at the term level.

**Table 2:** Mentions of health care system design ideas.

Attributes			Design idea overlay^a^						
			Universal health care		Medicare for All		Single payer		Socialized medicine
Definition^b^			A system where all citizens have access to health care services without financial hardship		A proposed system to expand the US Medicare program to cover all individuals, eliminating private insurance		A system where a single entity (usually the government) pays for all health care costs		A system where the government not only funds but also provides the health care services
Comments, N			3638; “universal health” or “universal healthcare”		2909; M4A or “medicare for all”		1474; “single payer” or “single-payer”		716; “socialized medicine” or “socialised medicine”
**Prevalence of comments that mention each design idea within a term-specific comment collection**									
	Highest-scoring term on a corresponding overlay	*Private room* (12/95, 12.6% of comments also mention universal health care)		*Warren* (116/276, 42% of comments also mention Medicare for All)		*Administrative cost* (16/108, 14.8% of comments also mention single payer)		*Medical innovation* (5/108, 4.6% of comments also mention socialized medicine)	
**Share of comments within ideological terms^c^**									
	*Socialism/socialist*	+1.44 SD		+0.04 SD		−0.16 SD		+0.64 SD	
	*Communism/communist*	+3.06 SD		−0.18 SD		−0.65 SD		+0.45 SD	
	*Capitalism/capitalist*	−0.33 SD		−0.28 SD		−0.49 SD		−0.53 SD	

^a^Interactive overlays are available from the left panel (view>items>color >) [42].

^b^Commenters defined health system design ideas in different ways and sometimes used them interchangeably. For example, some commenters talked generally about a state-managed health care system in reference to both single payer and socialized medicine.

^c^Normalized health system design idea overlay scores for 3 ideology nodes are shown relative to all nodes’ mean share of comments mentioning that specific health system design idea. Plus or minus signs refer to above or below all terms’ mean share, expressed in SD units, within each health system design idea overlay.

As shown in Table 2, the most frequently mentioned health system design idea in our comments—universal health care—was discussed in connection to *private room*, the highest-scoring term on the universal health overlay. The term *private room* also had above-average share (3/95, 3%) of comments, with at least 1 (6%) of 18 British-spelled words. US residents and foreign nationals discussed semiprivate and private hospital rooms as a desirable high standard for hospital stays. Commenters with experience in universal health systems explained that such systems serve everyone but may not provide extra luxuries unless a patient is also covered by private insurance or pays out of pocket. Several comments expressed preferences for universal health care systems with balanced public and private health care. Private rooms, marble floors, and hotel-like amenities in US hospitals were discussed as luxuries available to the rich, while care was being denied to the poor.

At the bottom of Table 2, we show 3 ideological terms and compare the extent to which they are linked to each health system design idea. For universal health overlay, the data address the following question: In node *socialism/socialist*, what is the share of comments that mentioned universal health and how far is this share, in SD units, away from the universal health care overlay’s mean for all nodes? Compared to 3 other concepts (Medicare for All, a single-payer system, and socialized medicine), universal health care was most strongly linked to discussions of communism and socialism. Specifically, the share of universal health care comments in the node *socialism/socialist* was much greater than that in most other nodes (1.44 SD above all terms’ mean). It was even higher for the node *communism/communist* (3.06 SD above the mean).

While discussing Medicare for All in early 2020, YouTube commenters were concerned that it was insufficiently supported by Elizabeth Warren, as compared to Bernie Sanders, which explains why *Warren* was the highest-scoring term in the Medicare for All overlay. In addition to questioning the political viability of Medicare for All, commenters expressed concerns about its funding and tax increases, possible loss of preferred private insurance, unemployment among health insurance workers, increased wait times, diminished quality of care, and fluctuating government or political control over reproductive health.

The highest-scoring term on the single-payer overlay, *administrative cost*, was often mentioned with a term *middleman*, an unnecessary intermediary, for example, private insurance companies and for-profit corporate interests. Discussions of single payer, administrative costs, and middlemen turned into debates. Advocates cited the potential for significant savings and increased efficiency by eliminating the profit-driven insurance model. They pointed to Medicare’s low overhead as evidence that a single-payer system could reduce administrative costs. By cutting out middlemen, single-payer systems bring down administrative costs and simultaneously simplify system navigation and transactions for patients, restrain profiteering, reduce health care fraud, and open health care systems to cost control. Critics, however, expressed skepticism about the efficiency of government-run systems, cautioning that replacing one bureaucratic structure with another may not achieve the expected reductions in administrative costs.

Finally, the term *medical innovation* had the highest share of comments that mentioned socialized medicine. The comments often referred to the United States’s top position in producing medical innovations. Several US commenters suggested that countries with socialized medicine rely upon US innovations without contributing comparable advancements in new treatments or medical technologies. US medical innovations, according to their comments, come at high cost but also contribute to high quality of care. Others expressed disagreement, saying the United States ranked fourth on medical innovation, behind Switzerland, Germany, and the Netherlands. In addition, hopes were expressed that rising costs of US health care could be controlled through medical innovations, especially in older adult care.

Of the 4 health system design ideas we analyzed, the concept of single-payer health system had the lowest use of ideological terms. The distribution of scores across the single-payer overlay shows that single-payer discussions were less prevalent in ideological terms (*socialism/socialist, communism/communist,* and *capitalism/capitalist)* than in other terms we mapped. In the *socialism/socialist* node, an above mean share of comments about Medicare for All (+0.04 SD), socialized medicine (+0.64 SD), and especially universal health care (+1.44 SD) indicated greater use of ideological terms, as compared to single-payer discussions (–0.16 SD). In addition, the universal health care discussion was much more centered around communism or communist (+3.06 SD) compared to the single-payer discussion (–0.65 SD).

## Discussion

### Overview

We discuss 2 sets of findings. First, we summarize our evaluation of the semantic network. We elaborate on the implications of repurposing VOSviewer to subsequent social media studies and anticipate scientific advances that may result from its broad application. Second, we summarize our US health system insights and discuss their policy implications, pointing out limitations.

### VOSviewer Term Co-Occurrence Network as a Social Media Analysis Method

VOSviewer is one of several programs available to researchers for conducting semantic network analysis. For example, previous studies have used the Fruchterman-Reingold algorithm [44], Gephi [45], and R [46] to build semantic networks. At the same time, VOSviewer’s user-friendly interface is suitable for users without advanced technical skills. Regardless of the tools used in their construction, semantic networks promise to represent knowledge, while their interconnected nodes likely capture meaning [12], as demonstrated by this analysis.

We used VOSviewer as a data visualization tool to respond to the critical need to decrypt chaotic and extensive social media discussions on a socially important topic. Our analysis suggests that VOSviewer produces visualizations with high information density, interactivity, and interpretive richness. In addition, we obtained evidence regarding the following characteristics of the VOSviewer-generated network: (1) robustness or resilience to variations in data, (2) content representativeness of the diversity of issues related to the US health system, (3) structural relevance defined as meaningful network relationships, and (4) semantic accuracy defined as accurate representation of comment meaning. Our evaluation of the network’s decision support usefulness is discussed in the US Health System Insights and Their Policy Implications section.

First, our limited test of robustness confirmed the network’s resilience to the removal of approximately 3% of repeated comments from our corpus. If such comments were retained, identical comments by just 1 social media user would have produced a user-specific map cluster about medical debt and bankruptcy. Striving to build a network reflective of broad conversations, we chose to remove it, but the comments we removed were relevant to the US health system. The person who posted them might have tried to express desperation or draw attention to the seriousness of medical debt.

Second, the network comprehensively covered 10 thematic video groups, representing the entire diversity of video content about the US health care system. In other words, comments from all video groups were represented within the network nodes. Third, we observed a meaningful cluster layout that, overall, could be intuitively interpreted. Structural relevance was confirmed by spatial arrangement of nodes in the network, where the proximity of nodes corresponded to the co-occurring nature of the semantic relationships observed in the text from which the nodes were derived. Moreover, the network’s structure aligned with existing knowledge, for example, ACA provisions. Forth, multiple checks confirmed that the mapped terms, including unexpected or ambiguous ones, captured the meanings of posts as well as their context.

### Anticipated Scientific Advances of the VOSviewer Application to Social Media Analyses

The VOSviewer’s term co-occurrence mapping method and their custom overlays can advance computational social sciences through informative, contextualized semantic networks. Natural language processing enables unbiased extraction of relevant terms, with an option of manual term screening. Revealing large patterns in extensive source data, VOSviewer “visual narratives” [47] can guide researchers to efficiently allocate their analytical resources as they explore salient patterns of societal importance embedded in “context or domain-specific knowledge” [48]. As such patterns involve network terms—nouns and noun phrases that occur in comments—researchers can strategically focus on the most promising subsets of extant data. In addition, VOSviewer-enabled semantic networks bring to light the interdisciplinary nature of social media studies. According to our cluster map, an in-depth analysis of public perceptions of the US health system calls for input from scholars in fields such as communication, economics, health care management, medicine, political science, public health, and others.

Clusters model thematic structure at a macro scale; overlays provide interpretive richness. The method we demonstrated here offers a valuable way for researchers to experience relationships embedded in source data, some of which are hard to document using conventional analyses. Chronological overlays that show video dates, comment dates, and lags in time between the first and the nth comment offer clues on how the discussion progressed over time, enabling a study of unfolding discourses. This is particularly relevant for data from social media platforms, which are “inherently longitudinal” [48]. With additional automation, it would be possible to create dynamic network visualizations that are updated in near–real time as new comments are posted.

Another benefit of semantic map overlays is that they foster cluster exploration and hypothesis testing by combining different data sources. For the YouTube platform, overlays may reflect characteristics of comments, YouTube video channels, videos themselves, or social media users’ channels. Therefore, visual overlays represent many opportunities for innovation and experimentation. For example, information excluded during term selection can be brought back in overlays. In this study, we removed geographical references from the cluster model’s nodes but created an overlay to highlight discussions with British spelling.

The method we demonstrated in this study can also enhance the value of qualitative research. Resource-intensive qualitative methods can be deployed strategically, guided by the grasp of larger patterns evident in semantic networks. Semantic networks can be contextualized and nuanced through qualitative coding. The qualitative codes can then be incorporated into custom-designed overlays, leading to new hypotheses and qualitative analyses. This iterative approach enables visualization-assisted qualitative inquiry.

Given these methodological strengths, we believe that VOSviewer-enabled semantic network analyses of social media data can advance social science research in the digital era. Thinking even broader, the proposed method can be applied across a variety of contexts and data sources, not limited to social media, and across different disciplines, such as computational humanities.

### US Health System Insights and Their Policy Implications

#### Overview

Health care debates unfold in both in real life and online spheres. We examined digital publics’ discourse about the US health care system in response to YouTube videos from right, center, and left media outlets. The YouTube platform allows purposeful selection of videos by varied media outlets on different aspects of an issue. We provided evidence that thematic diversity of videos was passed on to the commentary, opening a door to the policy-relevant analysis of diverse viewpoints. The YouTube platform has emerged as a space for heated debates, thoughtful ideas, misconceptions, and personal narratives in response to the US health care system.

Understanding the viewpoints by social media users provides valuable input for policy makers, health care professionals, and advocates aiming to shape effective reforms. The insights gleaned from the VOSviewer semantic network carry significant implications, which we grouped into 3 categories (concerns about the health care system, domestic and global interconnections in health care discussions, and informing change through key health care discourse insights).

#### Concerns About the Health Care System

The clusters shed light on a wide range of areas of concern within the US health care system, including those that are likely to be voiced by the public when politicians mention universal health care, Medicare for All, a single-payer system, and socialized medicine. The network analysis was helpful in estimating the use of ideological terms in discussions of various health system design ideas and identifying related concerns, for instance, about continued medical innovation or patients’ access to private hospital rooms. The ideology and society cluster terms, derived from politicized comments, reflect the entrenched ideological conflicts and capitalism-socialism dichotomies within the YouTube discourse about the US health care system.

We observed that comments in the health care workforce cluster, particularly those about staff shortages and burnout, received many likes. This pattern points to a widely shared perception of the urgent need to address challenges faced by nurses and other health professionals. If corroborated across time and other data sources, this sentiment may translate into public support for health care reforms that enhance workforce well-being, improve nurse-to-patient ratios, and support the essential role of health care workers in the system.

Online discussions also highlight ongoing debates about the balance between public and private health care services. Policy makers can use these insights to formulate strategies that optimize the strengths of both sectors, ensuring accessibility, affordability, and quality of care. In sum, a VOSviewer-generated semantic network with overlays shows promise as a decision support tool for policy makers.

#### Domestic and Global Interconnections in Health Care Discussions

Health care reforms should consider the broader societal and political context of the country to build sustainable and politically viable solutions. The health care discourse we described incorporated widespread debates about political ideologies, societal issues such as racism, and economic considerations. While many of these issues were domestic, there was also a significant international component. Terms such as *national insurance, government hospital, private system*, *free education, unemployment,* and *justice* represented 6% to 8% of comments with at least 1 British-spelled word from our list. In much smaller concentrations (2.5%-4%), British-spelled comments appeared in the wellness discussion (*nutrition, vegetable,* and *memory*) and conversations about tax break (or cut), social health care, and private insurance companies. Adding evidence in support of semantic accuracy, several terms extracted from a nonzero share of British-spelled comments (*national insurance* and *social health care*) described societies outside of the United States.

The presence of British-spelled words in our data indicated the global nature of US health care discussions, which is evident in international comparisons of prices and patient experiences. YouTube discussions offered opportunities for US social media users to learn about foreign health systems and explore their benefits, trade-offs, and foundational values. The information was conveyed not by experts or politicians but by laypeople who had encountered foreign systems as taxpayers and patients. Some informants lived in several countries and could compare multiple systems. Informed by global perspectives, the US public may shift its expectations, prompting politicians to incorporate best practices, for example, affordable drugs and predictable costs of emergency patient transportation, into reform initiatives. At the same time, both the public and policy makers stand to benefit from reexamining their own misconceptions and rigid ideological beliefs in light of successful health care models and practices in other countries.

#### Informing Change Through Key Health Care Discourse Insights

Our semantic network analysis provides insights into the topics that garner the most attention and engagement in ongoing discussions. Health care reforms can be supported by targeted public education and awareness campaigns addressing these key themes, fostering informed public discourse and encouraging active participation in the reform process. Accordingly, policy makers should continuously monitor public sentiments on platforms such as YouTube to inform dynamic, responsive health care policies that adapt to changing societal needs and concerns. Finally, leveraging user engagement patterns, particularly standardized likes and ongoing discussions, can establish effective feedback loops between policy makers and the public. Understanding which aspects of the discourse resonate most strongly with the public allows for the refinement of reform strategies. We provided empirical evidence of links between specific public opinions on health system designs and ideological discourse; comments about universal health care had a much higher use of ideological terms than discussions of single-payer health systems. Overall, the key takeaways drawn from the VOSviewer-generated semantic network analysis provide actionable insights for shaping reforms in health care, which are responsive, inclusive, and aligned with the diverse perspectives expressed by the public on digital platforms.

Finally, we share 2 observations on how VOSviewer maps may support evidence-based policy making and communicating with stakeholders. One consideration is the empirical rootedness of the information we mapped. Decision makers are more likely to accept and act upon information perceived as “evidence based” [48], for example, maps that display intuitively interpretable terms grounded in actual text. In the study by van der Voort et al [47] on big data, decision makers “wanted ‘stories to tell’ to feed public debate and highlight problems and opportunities,” favoring reports at higher resolutions. In our study, clusters communicated broad narratives about the public discourse of the US health system, while overlays enriched and contextualized interpretation of narratives, adding complexity and specificity.

How well decision makers with different levels of education can decode VOSviewer data visualizations remains to be tested. We anticipate that for most decision makers, the learning curve of interpreting maps will be less steep than that for statistical outputs with comparable informational value. While overlays provide a multidimensional understanding of the discourse, they may be harder to decode than clusters. At the same time, the interactive nature of VOSviewer Online is likely to add interest and user engagement, helping to translate research findings into informed decision-making and actionable policy measures.

### Limitations

While VOSviewer offers a powerful tool for visualizing and analyzing co-occurrence networks, the algorithm’s effectiveness is contingent on the initial selection of terms. The manual screening of a list of terms introduces a potential bias. In addition, the study is limited to English language YouTube comments, which may not fully capture the broader public discourse on health care.

Further research is warranted to validate and expand upon our results. Future studies could use other advanced natural language processing techniques to enhance the accuracy of term selection and clustering. Moreover, a multiplatform analysis that includes other social media platforms and online forums would provide a more comprehensive understanding of public sentiment and discourse surrounding health care.

## Appendix

Multimedia Appendix 1 Supplementary information on video and comment analysis.

## Abbreviations

ACA: Affordable Care Act
DMV: Department of Motor Vehicles
DNR: do not resuscitate

## Data Availability

All data are available in the main text or Multimedia Appendix 1. Map files can be downloaded from map URLs provided in Multimedia Appendix 1. Original YouTube comments (initial comments and first-level replies) can be accessed through YouTube using the video descriptions provided in Multimedia Appendix 1.
