# Supplementary material for: Visualizing YouTube Commenters’ Conceptions of the US Health Care System: Semantic Network Analysis Method for Evidence-Based Policy Making
Source: JMIR Infodemiology. 2025 Feb 11;5:e58227. doi: 10.2196/58227 (PMC11862770; doi:10.2196/58227)
Supplement: Multimedia Appendix 1 [file infodemiology_v5i1e58227_app1.doc]

## Visualizing YouTube Commenters’ Conceptions of the United States Health care System: A Semantic Network Method for Evidence-Based Policymaking, <https://infodemiology.jmir.org/2025/1/e58227/>

# Multimedia Appendix 1

## TABLE OF CONTENTS

[Supplementary Text 2](#__RefHeading___Toc190072811)

[1. Links to Visualizations Provided in the Main Text of the Article 2](#__RefHeading___Toc190072812)

[Figure 1 2](#__RefHeading___Toc190072813)

[Figure 2 2](#__RefHeading___Toc190072814)

[Figure 3 2](#__RefHeading___Toc190072815)

[Figure 4 3](#__RefHeading___Toc190072816)

[Figure 5 3](#__RefHeading___Toc190072817)

[2. Video Selection 3](#__RefHeading___Toc190072818)

[3. Characteristics of Video Groups 4](#__RefHeading___Toc190072819)

[4. Term Selection 4](#__RefHeading___Toc190072820)

[5. Overlay Construction and Measures 5](#__RefHeading___Toc190072821)

[6. Evidence of Network Robustness 6](#__RefHeading___Toc190072822)

[7. Thematic Video Overlays 6](#__RefHeading___Toc190072823)

[Supplementary Figures 8](#__RefHeading___Toc190072824)

[Figure S1 8](#__RefHeading___Toc190072825)

[Figure S2 9](#__RefHeading___Toc190072826)

[Figure S3 13](#__RefHeading___Toc190072827)

[Figure S4 14](#__RefHeading___Toc190072828)

[Figure S5 15](#__RefHeading___Toc190072829)

[Figure S6 16](#__RefHeading___Toc190072830)

[Figure S7 17](#__RefHeading___Toc190072831)

[Figure S8 18](#__RefHeading___Toc190072832)

[Figure S9 19](#__RefHeading___Toc190072833)

[Figure S10 20](#__RefHeading___Toc190072834)

[Supplementary Tables 21](#__RefHeading___Toc190072835)

[Table S1 21](#__RefHeading___Toc190072836)

[Table S2 22](#__RefHeading___Toc190072837)

[References 23](#__RefHeading___Toc190072838)

# Supplementary Text

## 1. Links to Visualizations Provided in the Main Text of the Article

Follow the links to Leiden University’s VOSviewer Online app to interactively explore network visualizations from Figures 1-5.

Click on individual nodes representing terms to view additional information, such as their attributes and connections. Use zoom controls to focus on specific areas of the map for a closer examination of details. For an explanation of Leiden University’s VOSviewer Online app user interface, see <https://app.vosviewer.com/docs/user-interface/>.

### Figure 1

A co-occurrence network of terms extracted from the commentary to 53 YouTube videos about the U.S. Health care system: a cluster map. Binary-counted terms that occur 60 times or more were mapped.

- An interactive map is available from Leiden University’s VOSviewer application: <https://app.vosviewer.com/?json=https%3A%2F%2Fdrive.google.com%2Fuc%3Fid%3D1prIJGroOYiIHyUHn5sTdliPdoJc5xnun>
- Short URL: <http://tinyurl.com/4v6nafs5>

### Figure 2

Overlays to Figure 1 for mean comment date (top) and ongoing discussions (standardized scores, bottom).

- Mean comment date: <https://tinyurl.com/2cgsq4rb>. A high-resolution image is provided below (Figure S4).
- Ongoing discussions (standardized scores): <https://tinyurl.com/2xjw8zfx>. A high-resolution image is provided below (Figure S5).

### Figure 3

A mean comment likes (standardized) overlay to Figure 1: <https://tinyurl.com/26bnlv5r>.

### Figure 4

Overlays to Figure 1 depicting the distributions of comments that mention “universal health” (top) and “Medicare for All” (bottom).

- Share of comments that mention “universal health”: <https://tinyurl.com/226kyk6y>. A high-resolution image is provided below (Figure S7).
- Share of comments that mention “Medicare for All”: <https://tinyurl.com/28yyvf6u>. A high-resolution image is provided below (Figure S8).

### Figure 5

Overlays to Figure 1 depicting the distributions of comments that mention “single payer” (top) and “socialized medicine” (bottom).

- Share of comments that mention “single payer”: <https://tinyurl.com/2bc9dgyu>. A high-resolution image is provided below (Figure S9).
- Share of comments that mention “socialized medicine”: <https://tinyurl.com/226ubqzv>. A high-resolution image is provided below (Figure S10).

## 2. Video Selection

Video inclusion criteria

- Uploaded to YouTube between 2014 and 2023
- Watched at least 100K times
- Must have at least 800 comments as of August 28, 2023
- A channel that belongs to a news, educational, or entertainment organization
- Entire spectrum of U.S. health care, no restriction

Video exclusion criteria

- A channel that belongs to an individual
- Main focus is the Covid-19 pandemic

Video Search strategy

- Search terms: US health care (health care) system, health care (health care) in the US
- Searched YouTube suggestions when an eligible video was retrieved
- Searched most common channels for additional videos

## 3. Characteristics of Video Groups

We summarized characteristics of video groups in Tables S1 and S2.

Media outlets were divided into right (1), center (2), and left (3), using a simplified scoring system of AllSides Editorial Review, which was described as follows,

“During an AllSides Editorial Review, a panel of reviewers — which includes at least six people from the left, center, and right — reviews news reports from a source from the past six months to the present day, looks for types of media bias, deliberates, and comes to a general consensus on the source's bias. During a Small Group Editorial Review, a smaller panel of reviewers — one each from the left, center, and right — reviews and rates news content” [1].

We set a minimum of 800 comments per video and 100,000 views as our inclusion criteria for YouTube videos. We aimed to collect comments to videos from many different media outlets. It was not possible to achieve a perfect balance of videos from the left, center, and right because videos by right media outlets tended to have less views and comments than those on the left. Media outlets from the left accounted for 52% of videos (28 videos and 120,858 comments), followed by center (17 videos and 49,517 comments), and right (8 videos and 14,404 comments). Even though there was a relatively small number of comments evoked by videos from the right, we found many conservative voices in discussions elicited by left and center media.

## 4. Term Selection

Pre-established term exclusion criteria and human judgment guided our selection of terms from a list of 1,948 terms extracted by VOSviewer from YouTube comments. To limit noise and enhance conceptual clarity, we removed general terms (e.g., *advice*), references to time (e.g., *last year*) and geographical locations, as well as body parts, which accompanied explanations of medical procedures and illnesses, and terms with multiple meanings, such as *bill*, which was used in reference to both hospital billing and the legislative process.

We also removed terms that were very frequent and therefore obscured smaller terms (e.g., *government*), had lower value in communicating network’s thematic content (e.g., swear, mentions of family members), keeping in mind that some of these could be more effectively displayed as a network overlay (e.g., *Medicare for All,* which we chose not to merge with M4A that did occur more than 60 times and appeared in our network). When the meaning of the term was unclear, we took deep dives into the original data to understand how YouTube commenters used the term.

Human judgment was supplemented by two automations, a VOSviewer-calculated relevance score and network visualization. Highly relevant terms tended to be more specific to the U.S. health care system discussion than terms with low relevance scores. The default option in VOSviewer is to exclude 40% of the terms that score the lowest on relevance, however, we initially opted to manually override this default to maintain full control over the term selection. In addition to relevance scores, we examined term meaning in connection to other terms in our network. This strategy was especially useful later in the term selection process, when the most obvious exclusions were made but the network size had to be reduced for optimal comprehension.

In addition, we merged synonyms, e.g., *socialism* and *socialist*. We created a VOSviewer thesaurus file to exclude and merge terms, an audit trail of network modifications. Our thesaurus-cleaned final term list had 539 terms, which we mapped using a binary counting method and allowing VOSviewer’s algorithm to remove approximately 40% of terms with lowest relevance scores, resulting in 323 terms we mapped.

## 5. Overlay Construction and Measures

The overlay visualization is explained in section 3.1.2 of the VOSviewer manual [2]. Overlays are constructed with the help of a scores file, described in section 4.2.2 of the manual [2].

*Video Themes*. As shown in tables S1 and S2, videos were categorized into 10 groups based on their titles, short YouTube descriptions, and content: health care costs and financial issues (13 videos), health care policies and politics (9 videos), ACA/Obamacare reform (8 videos), health care systems in different countries (8 videos), health care workforce (7 videos), end-of-life health care (3 videos), single payer health care (2 videos), comedy on the U.S. health care (1 video), children’s health care (1 video), and Medicare for All by J. Oliver (1 video). We created a separate overlay for each video group. Posts to any of the videos within a group were coded as 1 and all other comments were coded as 0. Assigned at the term or node level, overlay colors reflected the average score, calculated as a mean of 0, 1 codes for all comments that mentioned the node’s term. A term score, multiplied by 100, corresponds to the percentage of comments in a node that were elicited by a video group.

*Comments with select British spellings*. We scored 18 words, aiming to identify terms that come from comments with higher and lower rate of British-spelled words. Spelling common in the United Kingdom or in the Commonwealth was detected in multiple clusters but the highest scoring terms were in cluster 2 (national insurance, government hospital, private system) and cluster 3 (free education, unemployment, justice). Each of these terms represented 6-8% of comments with at least one British spelled word from our list. In much smaller concentrations (2.5-4%), British-spelled comments appeared in the wellness discussion (nutrition, vegetable, memory) of cluster 1 and cluster 4 conversations about tax break (or cut), social health care, and private insurance companies.

*Mean Comment Date*. The time of comment’s posting on the YouTube platform was converted into a year with decimal points representing months and days since the start of the year. A term score reflects the mean year of all comments from which the term was extracted.

*Ongoing Discussions, Standardized and Unstandardized*. This measure reflects lags in time between the first and the *n*th comment. We computed time difference between the comment and the first comment to the same video, the averaged that for all comments from which a term was drawn. We had two measures, standardized and non-standardized. Standardized scores were calculated using the base-10 logarithm to account for skewed data, then normalized so that the mean is zero and scale points are standard deviations. Unstandardized scores reflect a fraction of a year. High scores indicate comments contributed long after the respective videos were posted. A standardized term score is the mean SD of all comments from which the term was extracted. The unstandardized term score is the mean time expressed as a fraction of a year.

*Mean Comment Likes, Standardized*. Each comment’s standardized likes were calculated using the base-10 logarithm to account for skewed data, then normalized so that the mean is zero and scale points are standard deviations. A term score reflects the mean SD of all comments from which the term was extracted.

*Comments by non-U.S. YouTube Users*. We used a list of 18 words with spelling common in the UK or in the Commonwealth to identify and code comments that used at least one of the words (coded as 1) and comments that did not (coded as 0). The words were defence, favour, labour, apologise, recognise, humour, paediat*, offence, organisation, queue, travelling, favourite, honour, centre, programme, privatisation, and neighbour. A term score, multiplied by 100, corresponds to the percentage of comments in a node that contained one of the abovementioned words.

*System Design Ideas*. Comments that mentioned universal health* were coded as 1 or 0 if they did not mention it. A term score, multiplied by 100, corresponds to the percentage of comments in a node that contained language consistent with universal health care. The same process was repeated for Medicare for All, single payer, and socialized medicine.

## 6. Evidence of Network Robustness

In Figure S1, cluster 9 is represented by nodes that were extracted from comments contributed by a single YouTube user. This cluster was not replicated when 5,575 duplicate comments were excluded from the map. The rest of the network structure was retained, although the number of clusters was greater due to a higher resolution.

## 7. Thematic Video Overlays

In designing video overlays, we took into account that the commentary originated within the broader framework of the YouTube platform design, where videos uploaded by channel owners drove user engagement with the video content, giving rise to the commentary. Considering this unique aspect in the design of the YouTube platform, it was important to understand social media users’ comments within the context of the videos that elicited them.

A thematic analysis of video titles and descriptions led us to categorize 53 videos into 10 groups: health care costs and financial issues (13 videos), health care policies and politics (9 videos), ACA/Obamacare health care reform (8 videos), health care systems in different countries (8 videos), health care workforce (7 videos), end-of-life health care (3 videos), single payer health care (2 videos), comedy on American health care (1 video), children’s health care (1 video), and Medicare for All by John Oliver (1 video).

A separate overlay was added for each video group (Figure S2). Posts to any of the videos within a group were coded as 1 and all other comments were coded as 0. Assigned at the node level, overlay colors reflected the average score, calculated as a mean of 0, 1 codes for all comments that mentioned the node’s term. The node score, multiplied by 100, corresponds to the percentage of comments in a node that were elicited by a video group.

Comments differed on which videos elicited them and each node differed in how many comments and which specific comments it represented. Consequently, the overlay visualized intricate relationships between the commentary and the material that evoked it. The health care systems in different countries overlay helps to illustrate this idea.

# Supplementary Figures

## Figure S1

A co-occurrence network (cluster map) of terms extracted from 184,768 comments, including 5,575 duplicate comments. Binary-counted terms that occur 60 times or more were mapped.


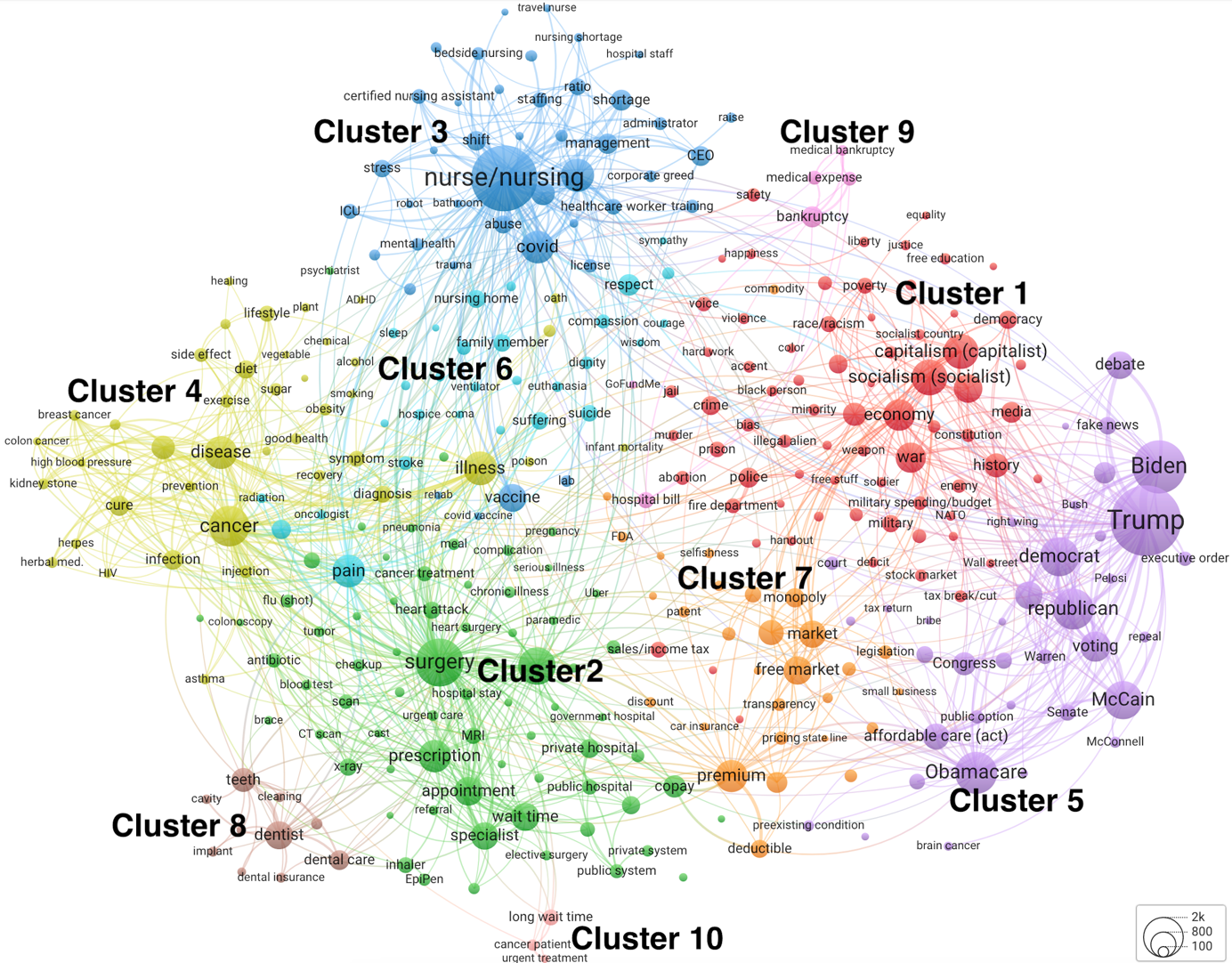


## Figure S2

Overlays to Figure 1 depicting the distributions of comments from 10 thematic video groups.

Follow links to Leiden University’s VOSviewer Online app to interactively explore these visualizations:

1. Health care costs and financial issues (13 videos): <https://tinyurl.com/2944le62>
2. Health care policies and politics (9 videos): <https://tinyurl.com/23omf35k>
3. ACA/Obamacare reform (8 videos): <https://tinyurl.com/22gkt4f6>
4. Health care systems in different countries (8 videos): <https://tinyurl.com/2cph2lwf>
5. Health care workforce (7 videos): <https://tinyurl.com/2dl2kjop>
6. End-of-life health care (3 videos): <https://tinyurl.com/222anwnx>
7. Single payer health care (2 videos): <https://tinyurl.com/282tee6j>
8. Comedy on the US health care (1 video): <https://tinyurl.com/2aaa3kjk>
9. Children’s health care (1 video): <https://tinyurl.com/2736oyvk>
10. Medicare for All video by John Oliver (1 video): <https://tinyurl.com/28h3ysel>

See next page.

| A. Cluster map (reference) | B. Health care costs and financial issues |
| --- | --- |
| 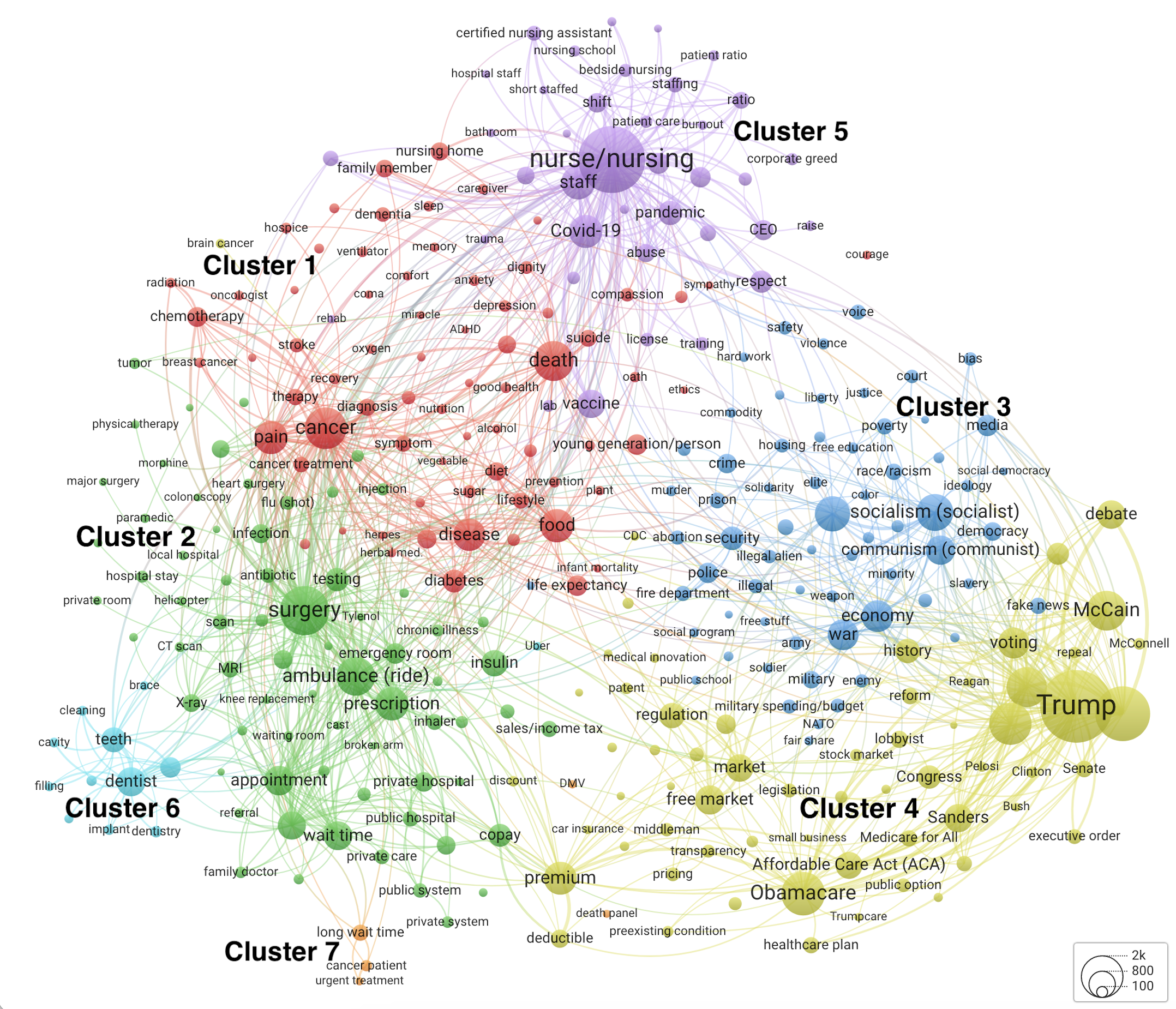 | 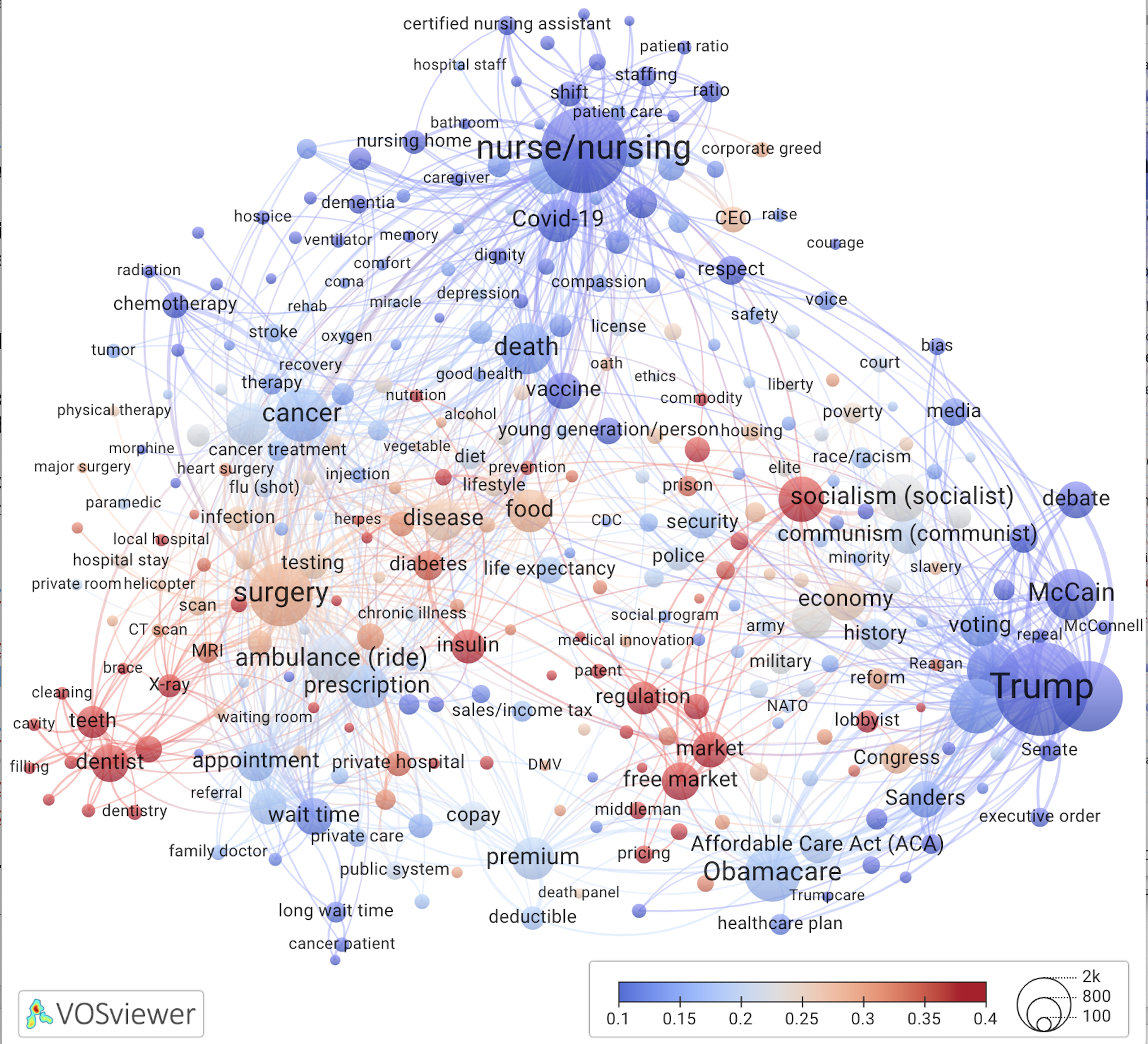 |
|  |  |
| C. Health care policies and politics | D. ACA/Obamacare health care reform |
| 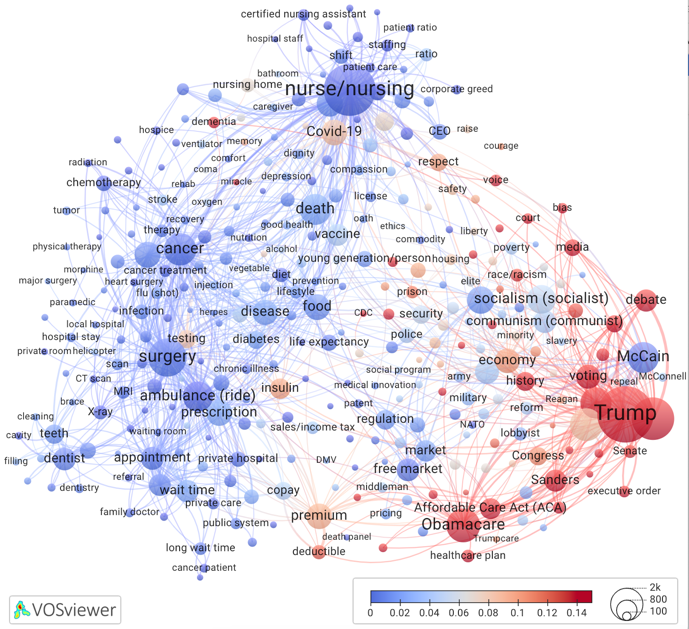 | 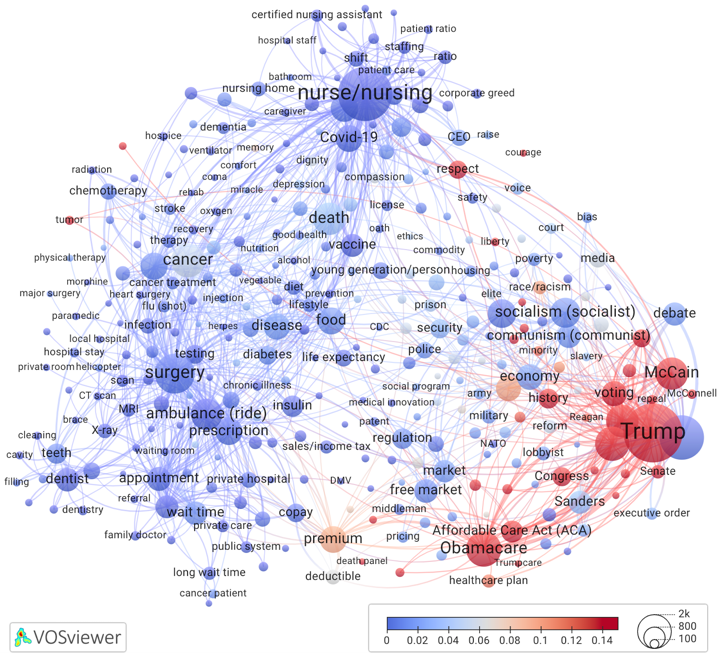 |

| E. Health care systems in different countries | F. Health care workforce |
| --- | --- |
| 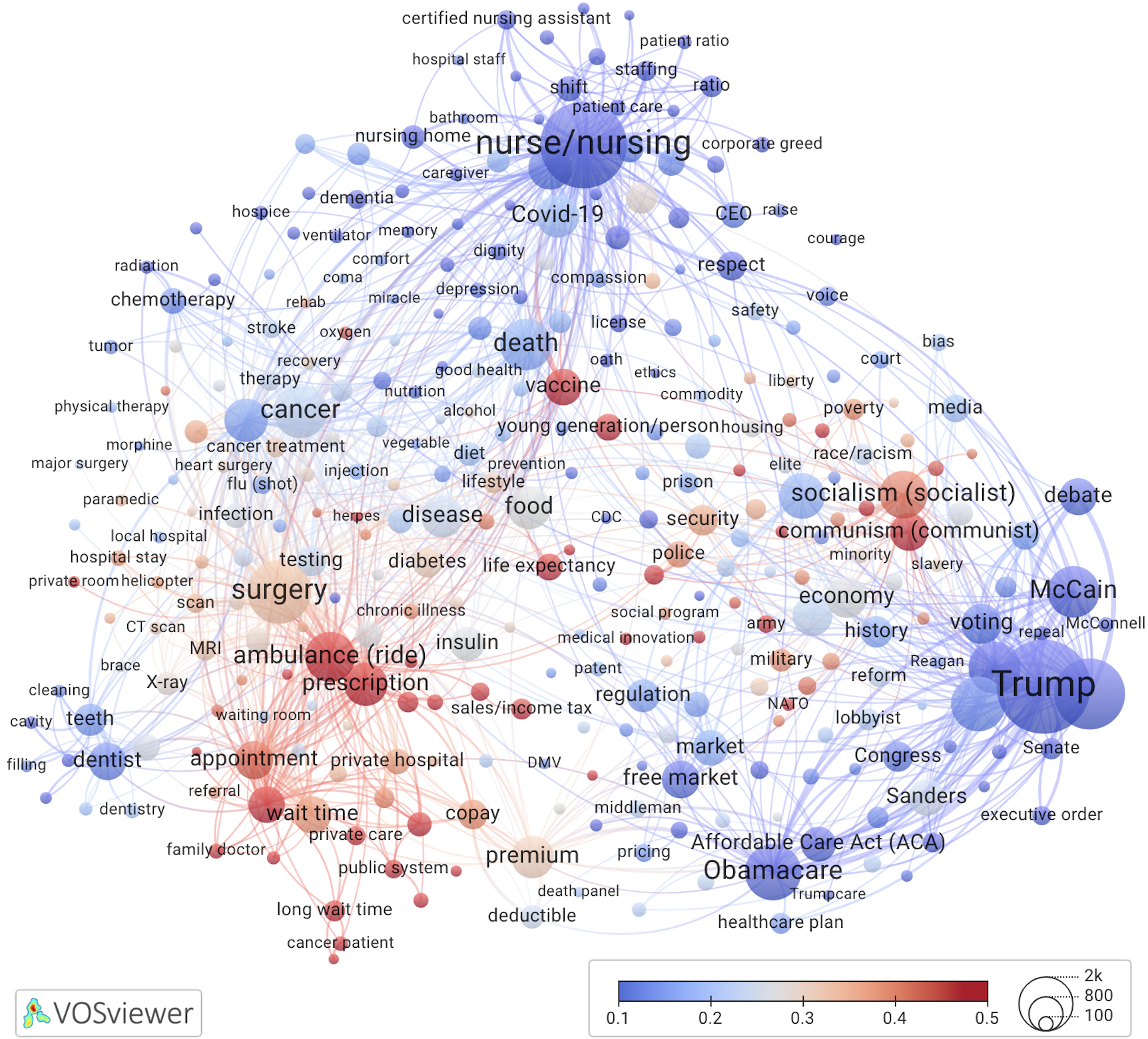 | 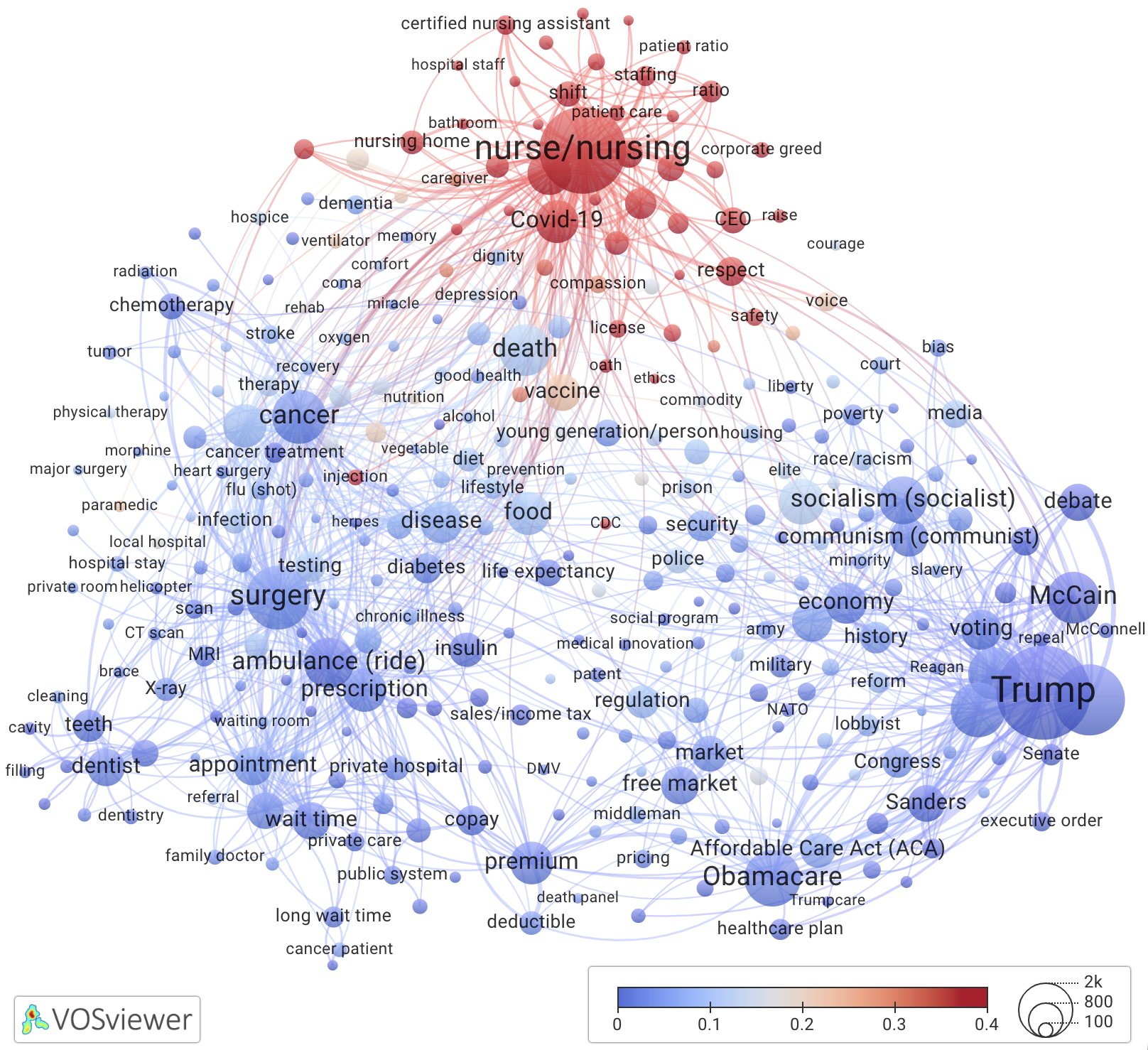 |
|  |  |
| G. End-of-life health care | H. Single payer health care |
| 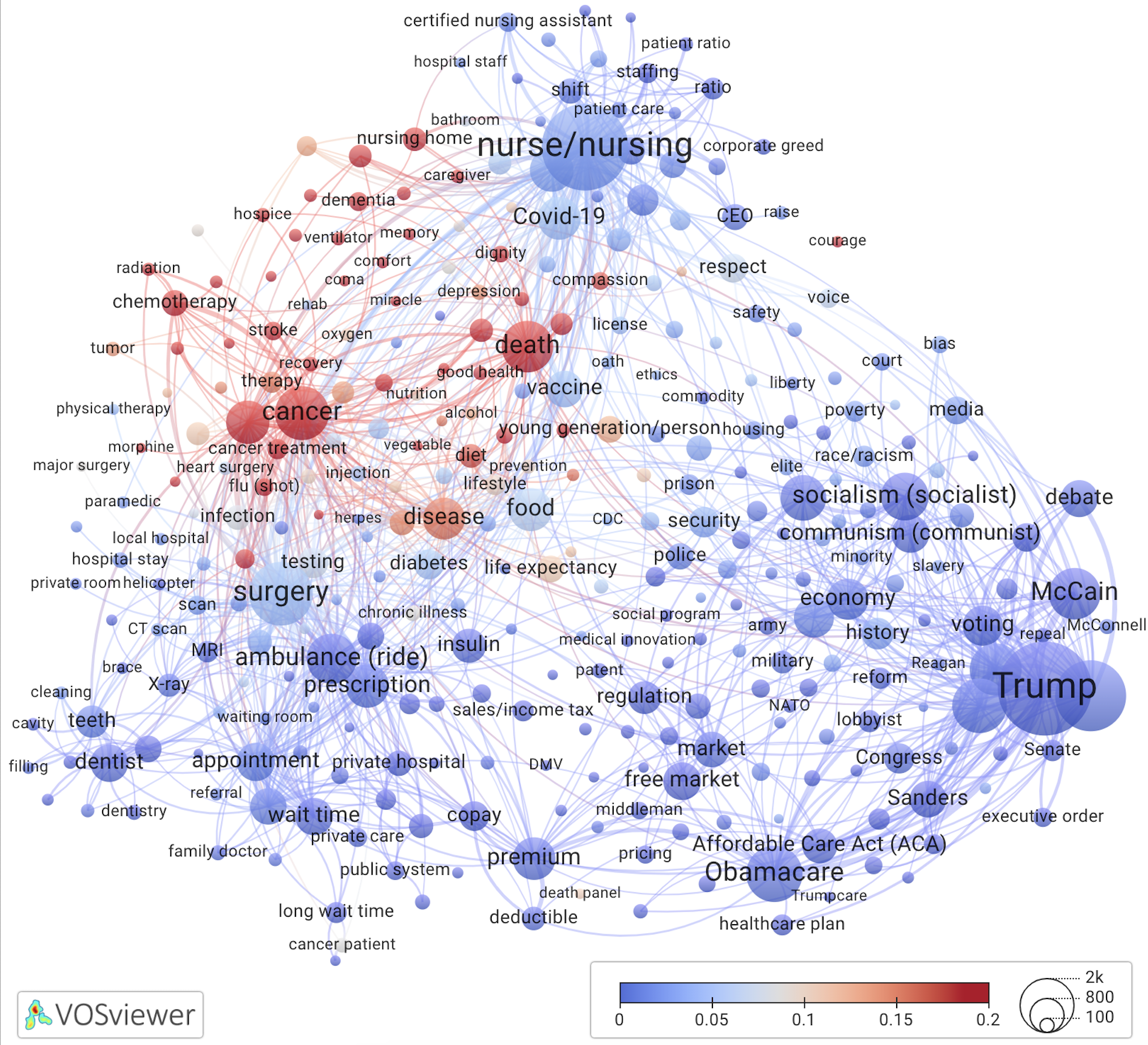 | 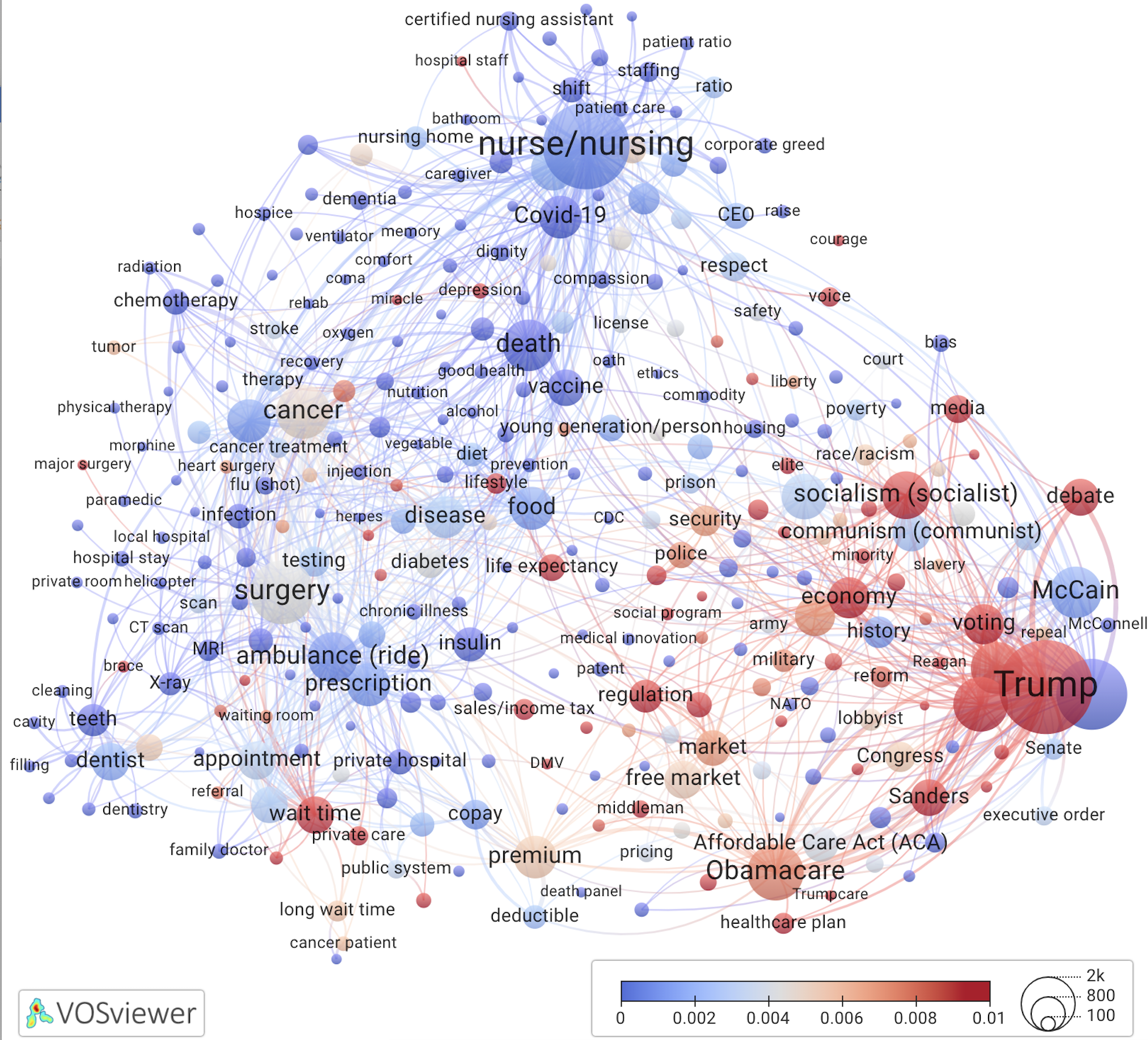 |

| I. Children’s health care | J. Comedy on American health care |
| --- | --- |
| 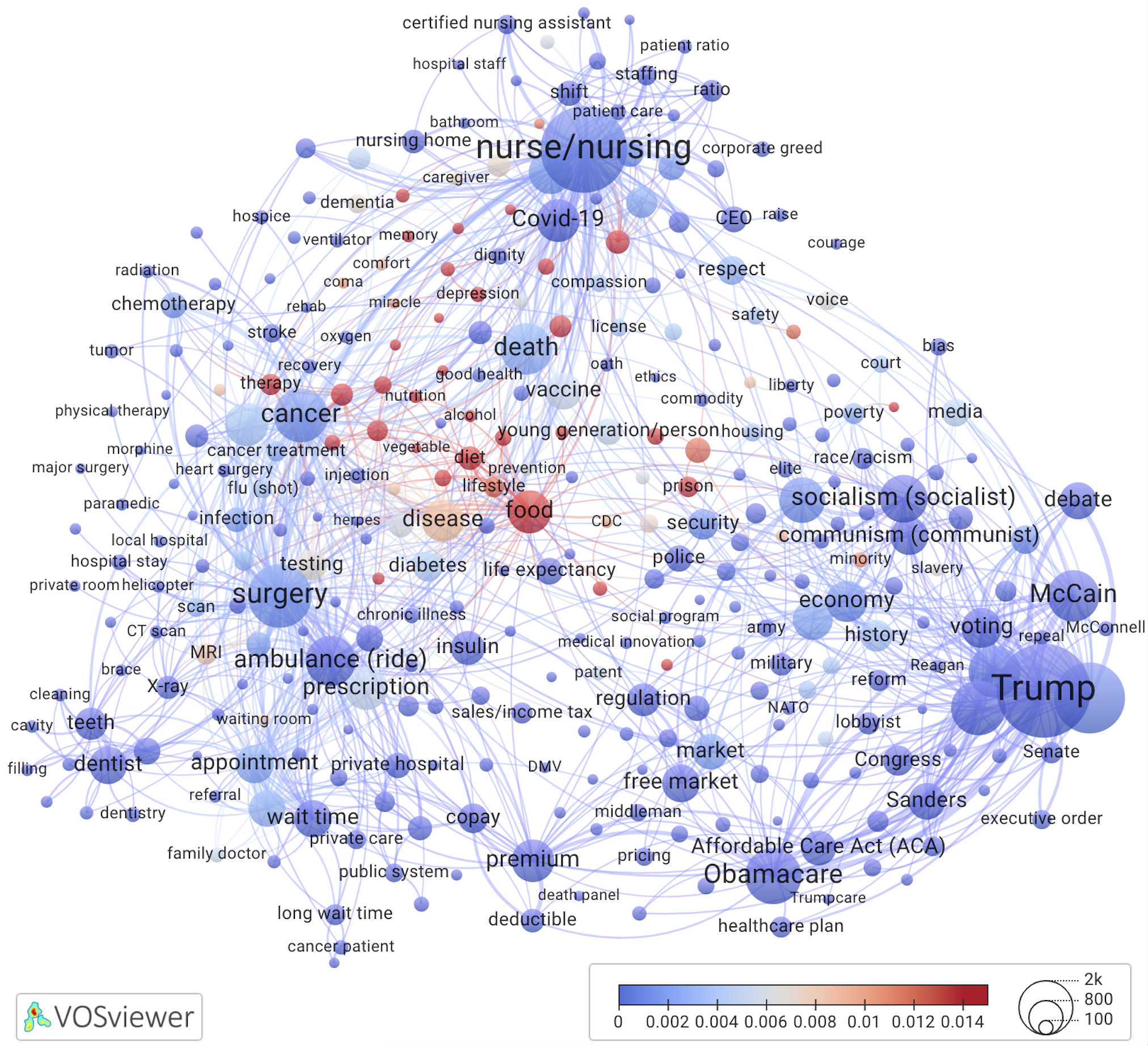 | 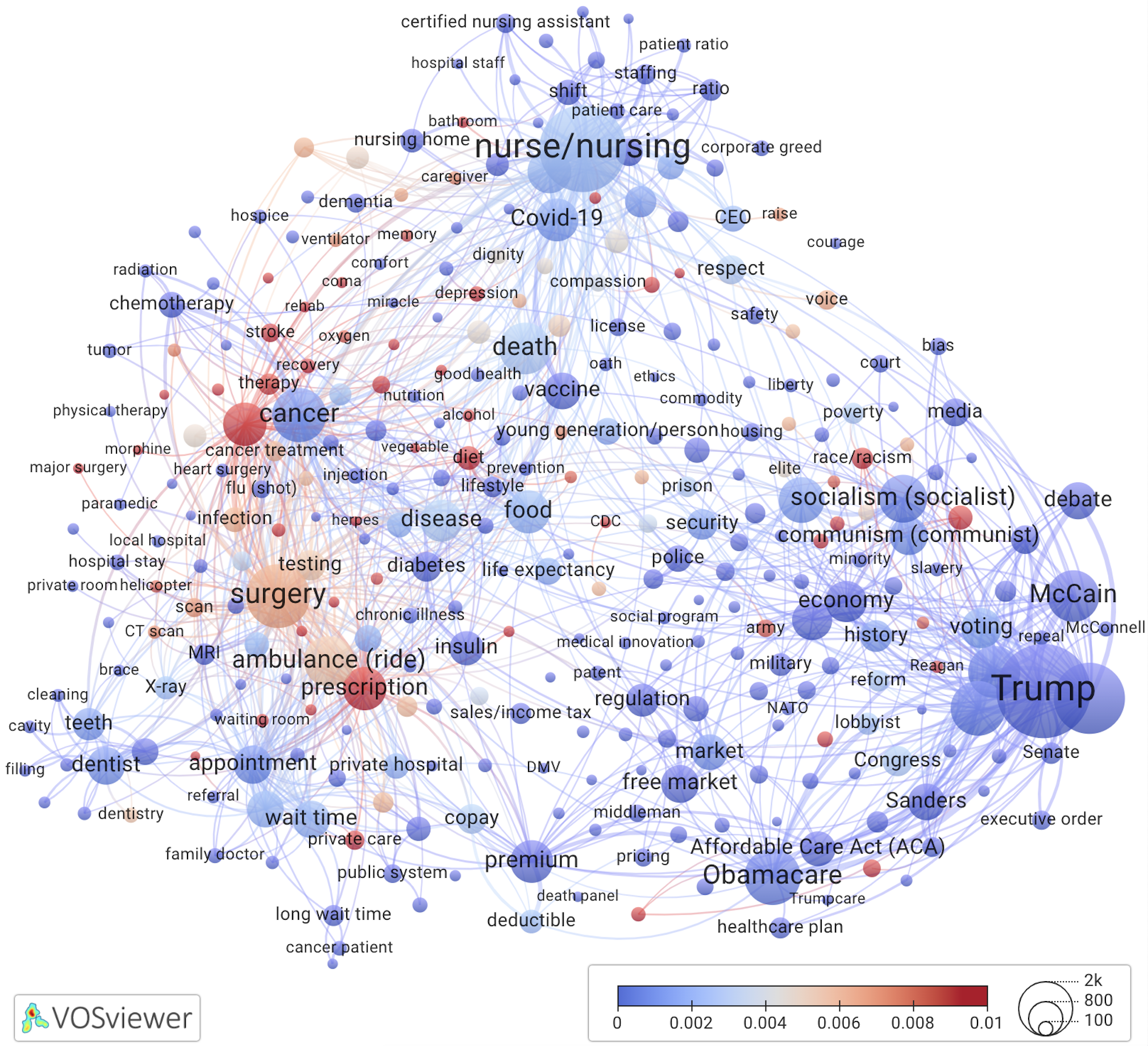 |
|  |  |
| K. Medicare for All video by John Oliver | L. Cluster map (reproduced from above for easy reference) |
| 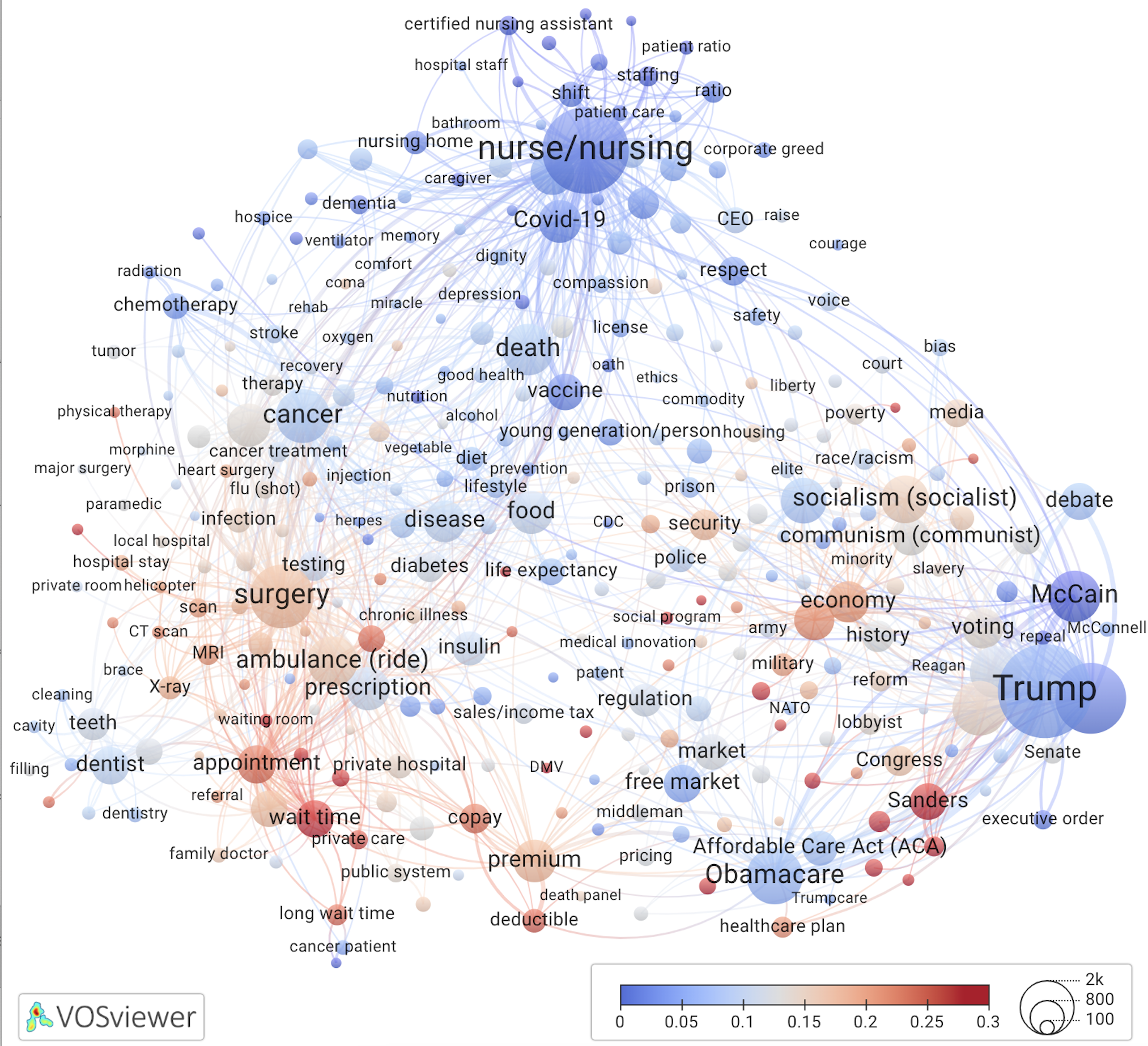 | 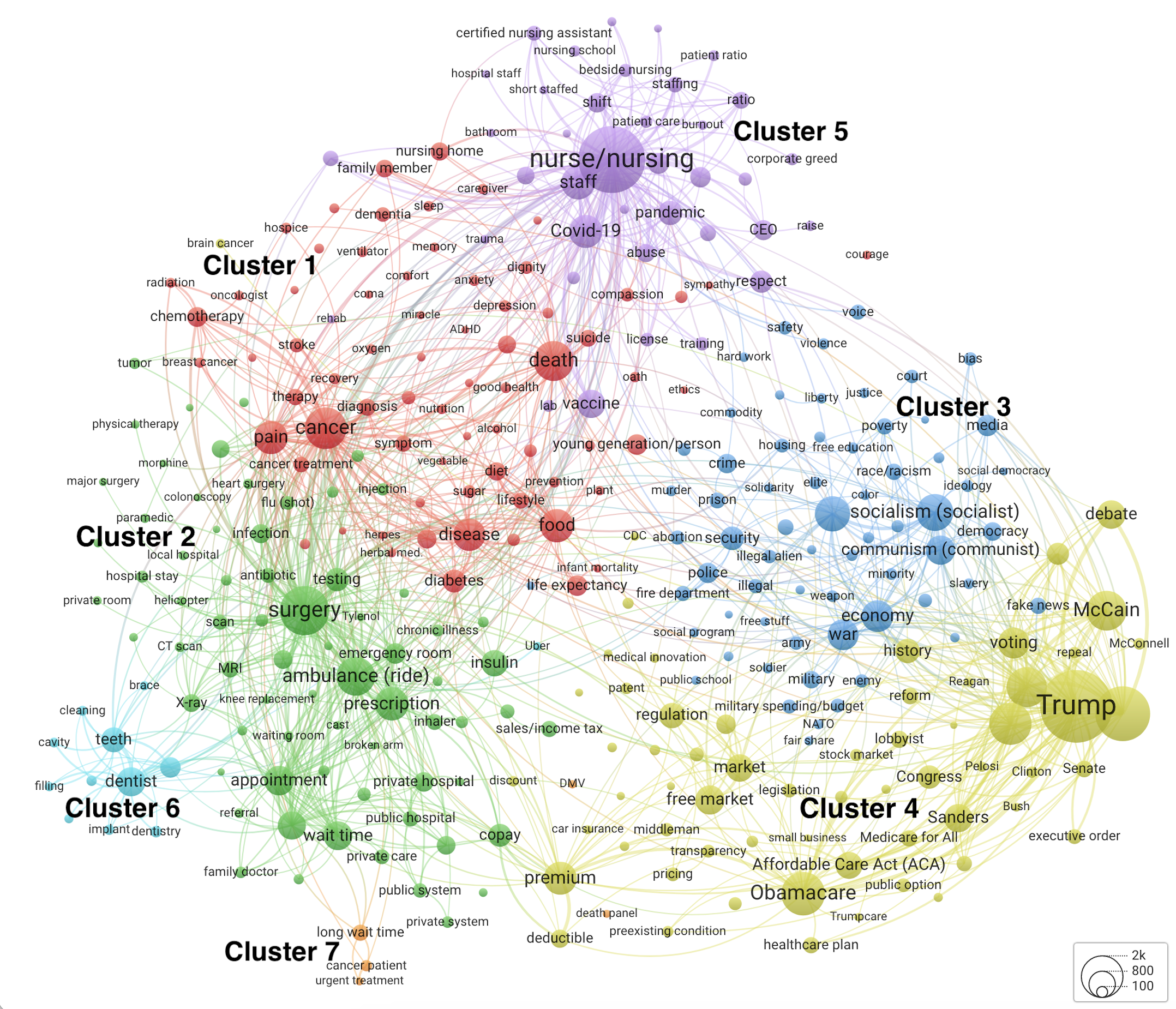 |

## Figure S3

An overlay to Figure 1 for ongoing discussions (unstandardized time calculated as a fraction of a year). Follow a link to Leiden University’s VOSviewer Online app to interactively explore this visualization: <https://tinyurl.com/29vvh3je>.


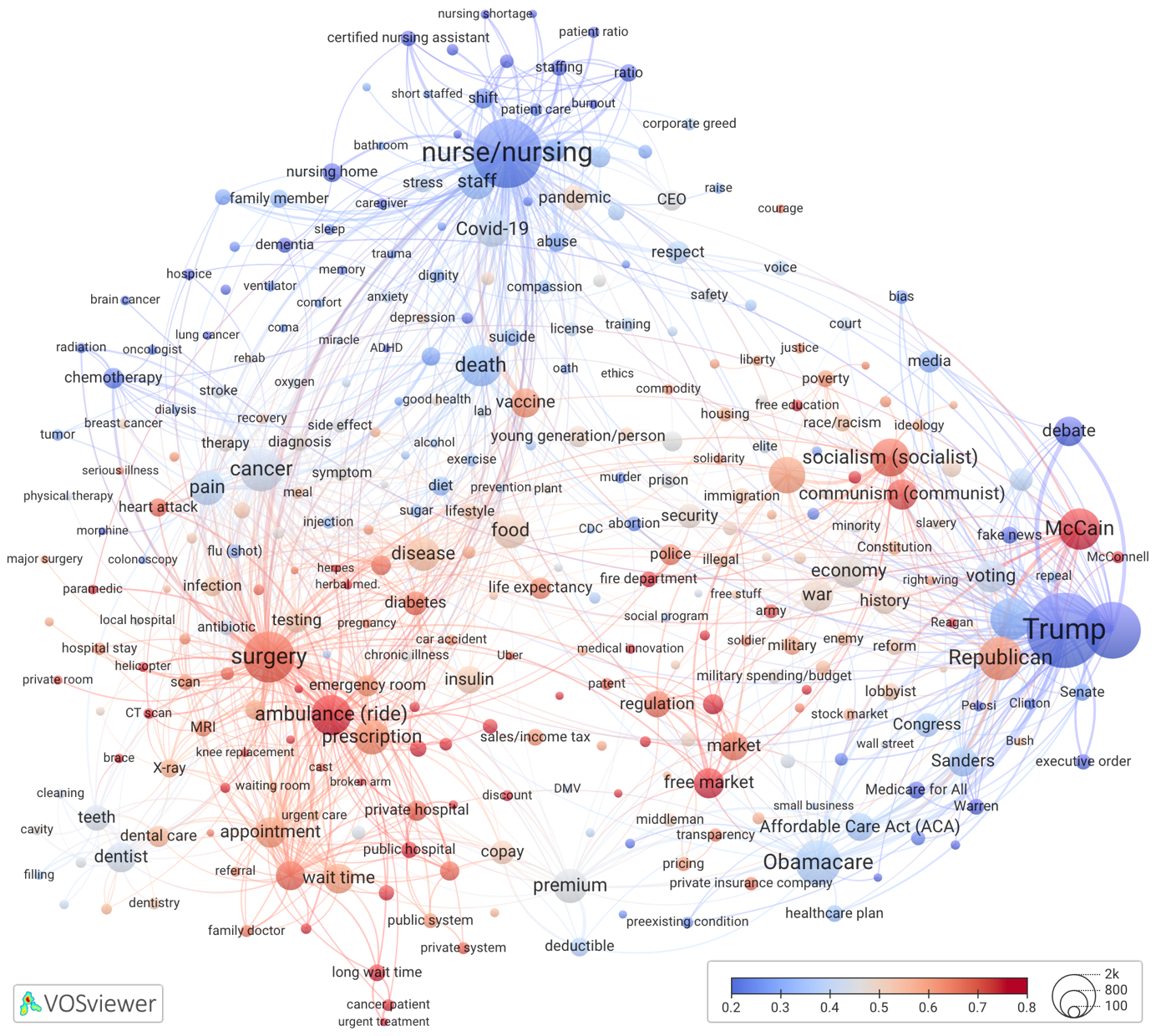


## Figure S4

A high-resolution image of an overlay to Figure 1 for mean comment date. Follow a link to Leiden University’s VOSviewer Online app to interactively explore this visualization: <https://tinyurl.com/2cgsq4rb>.


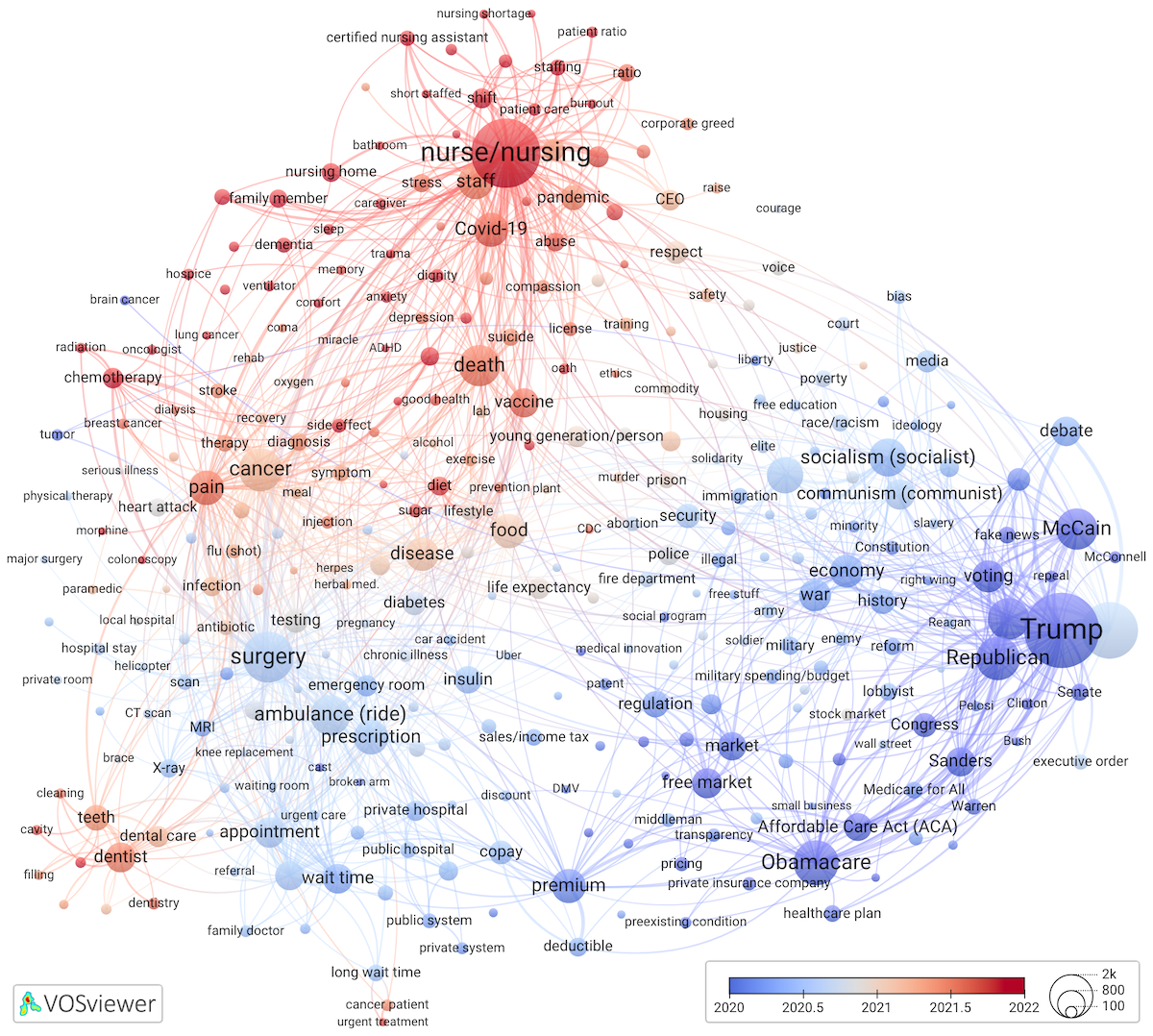


## Figure S5

A high-resolution image of an overlay to Figure 1 for ongoing discussions (standardized scores). Follow a link to Leiden University’s VOSviewer Online app to interactively explore this visualization: <https://tinyurl.com/2xjw8zfx>.


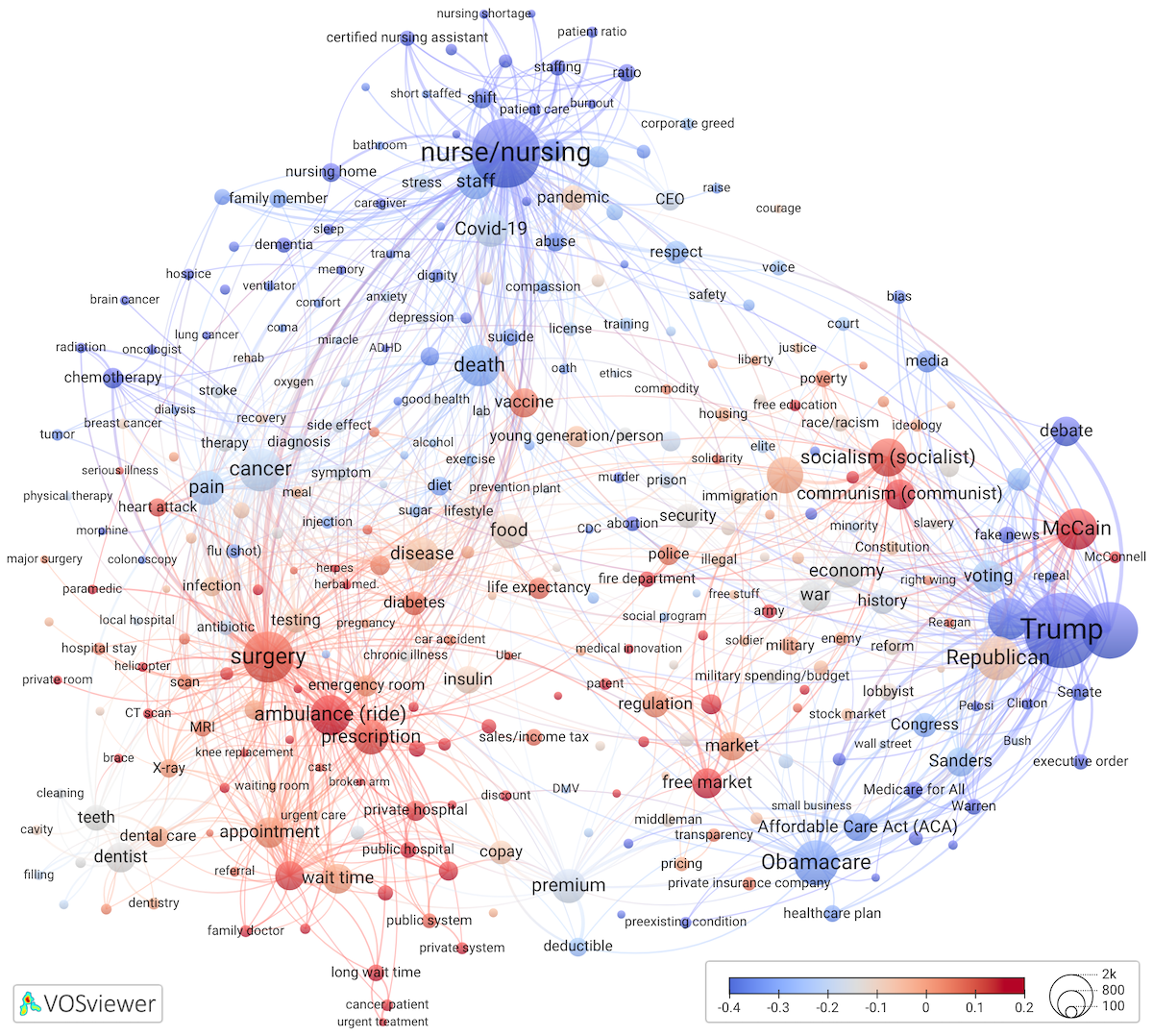


## Figure S6

An overlay to Figure 1 for comments with select British spellings. Follow a link to Leiden University’s VOSviewer Online app to interactively explore this visualization: <https://tinyurl.com/267vbma5>.


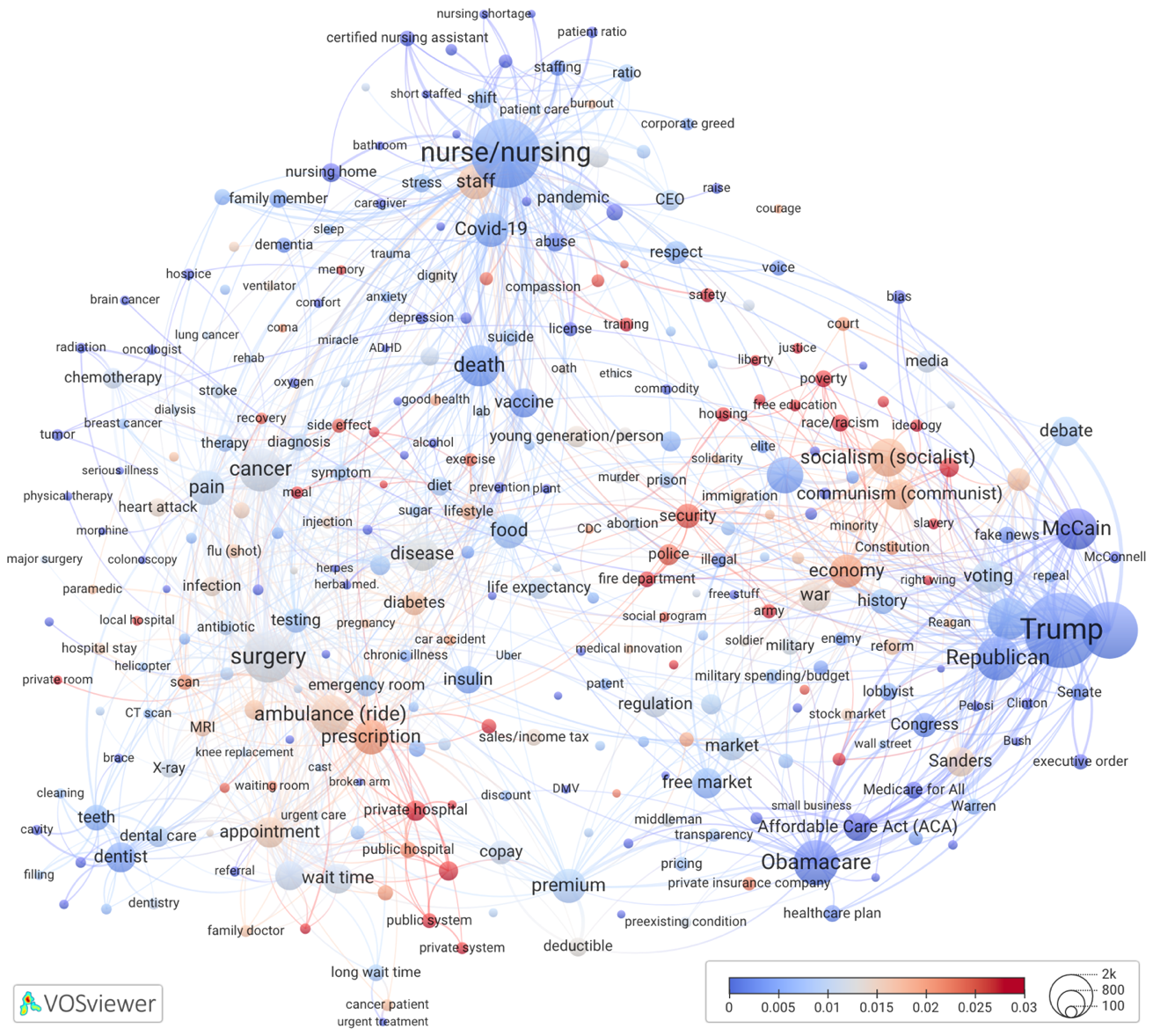


## Figure S7

A high-resolution image of an overlay to Figure 1 depicting the distribution of comments that mention “universal health.” Follow a link to Leiden University’s VOSviewer Online app to interactively explore this visualization: <https://tinyurl.com/226kyk6y>.


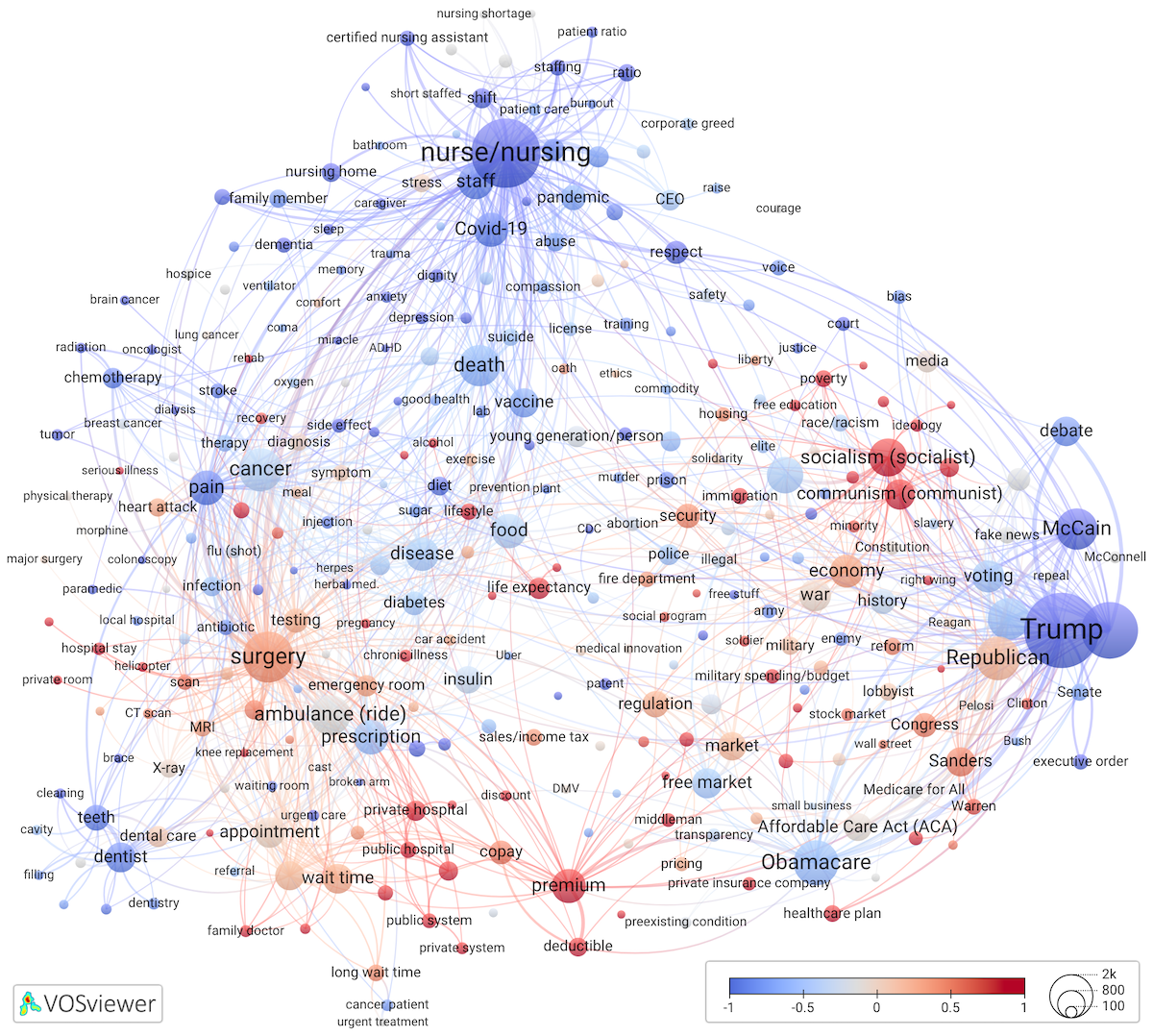


## Figure S8

A high-resolution image of an overlay to Figure 1 depicting the distribution of comments that mention “Medicare for All.” Follow a link to Leiden University’s VOSviewer Online app to interactively explore this visualization: <https://tinyurl.com/28yyvf6u>.


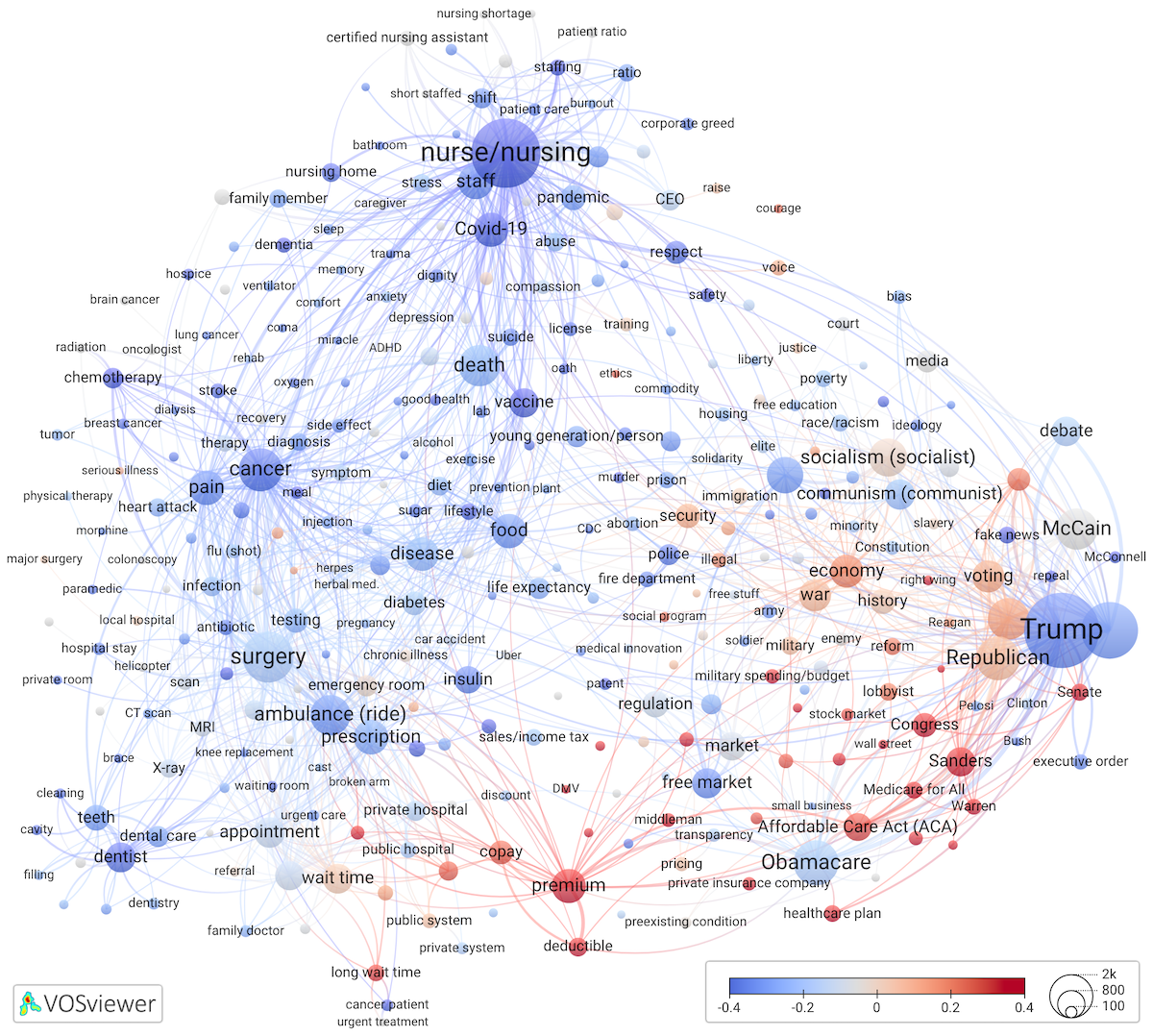


## Figure S9

A high-resolution image of an overlay to Figure 1 depicting the distribution of comments that mention “single payer.” Follow a link to Leiden University’s VOSviewer Online app to interactively explore this visualization: <https://tinyurl.com/2bc9dgyu>.


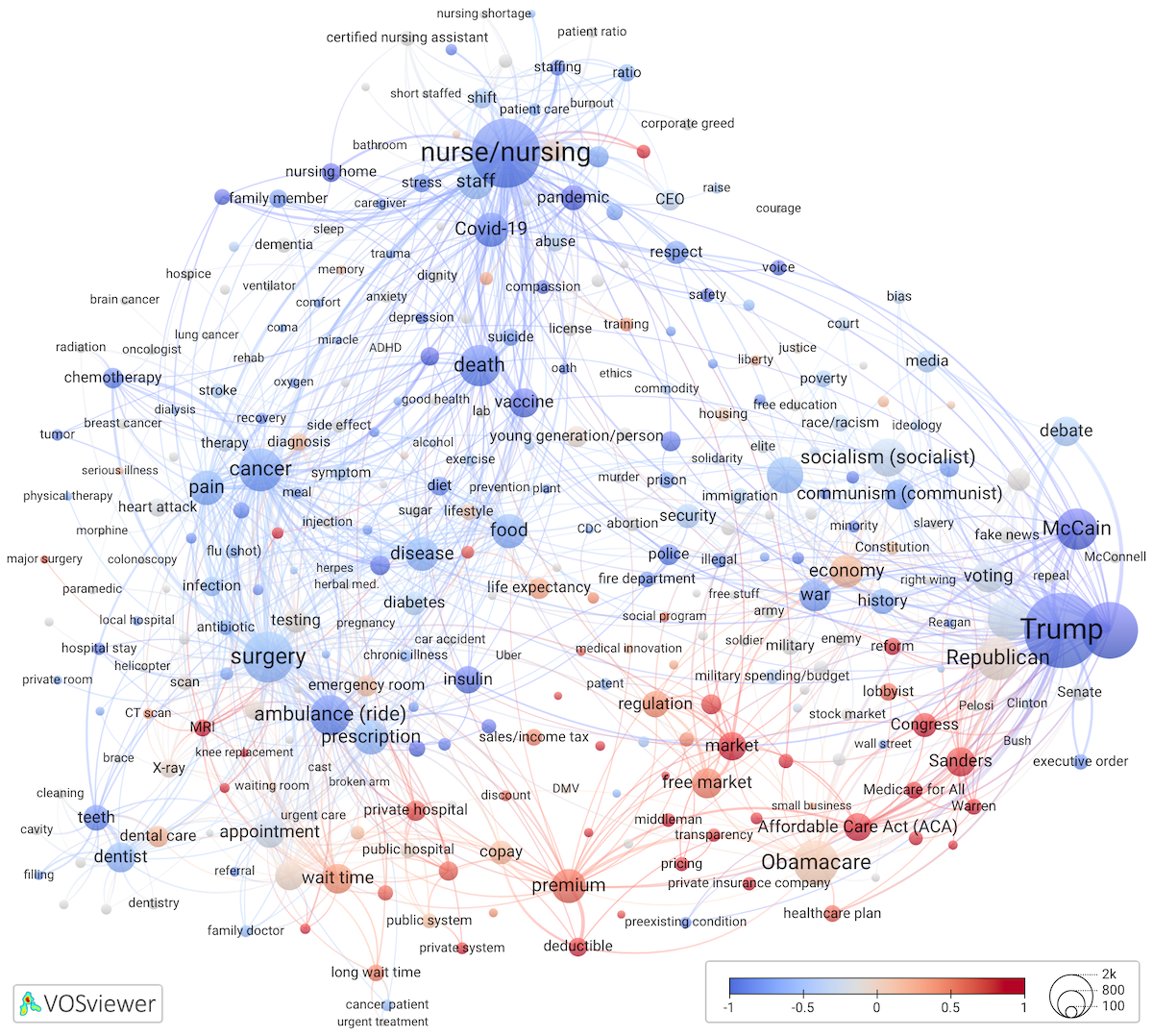


## Figure S10

A high-resolution image of an overlay to Figure 1 depicting the distribution of comments that mention “socialized medicine.” Follow a link to Leiden University’s VOSviewer Online app to interactively explore this visualization: <https://tinyurl.com/226ubqzv>.


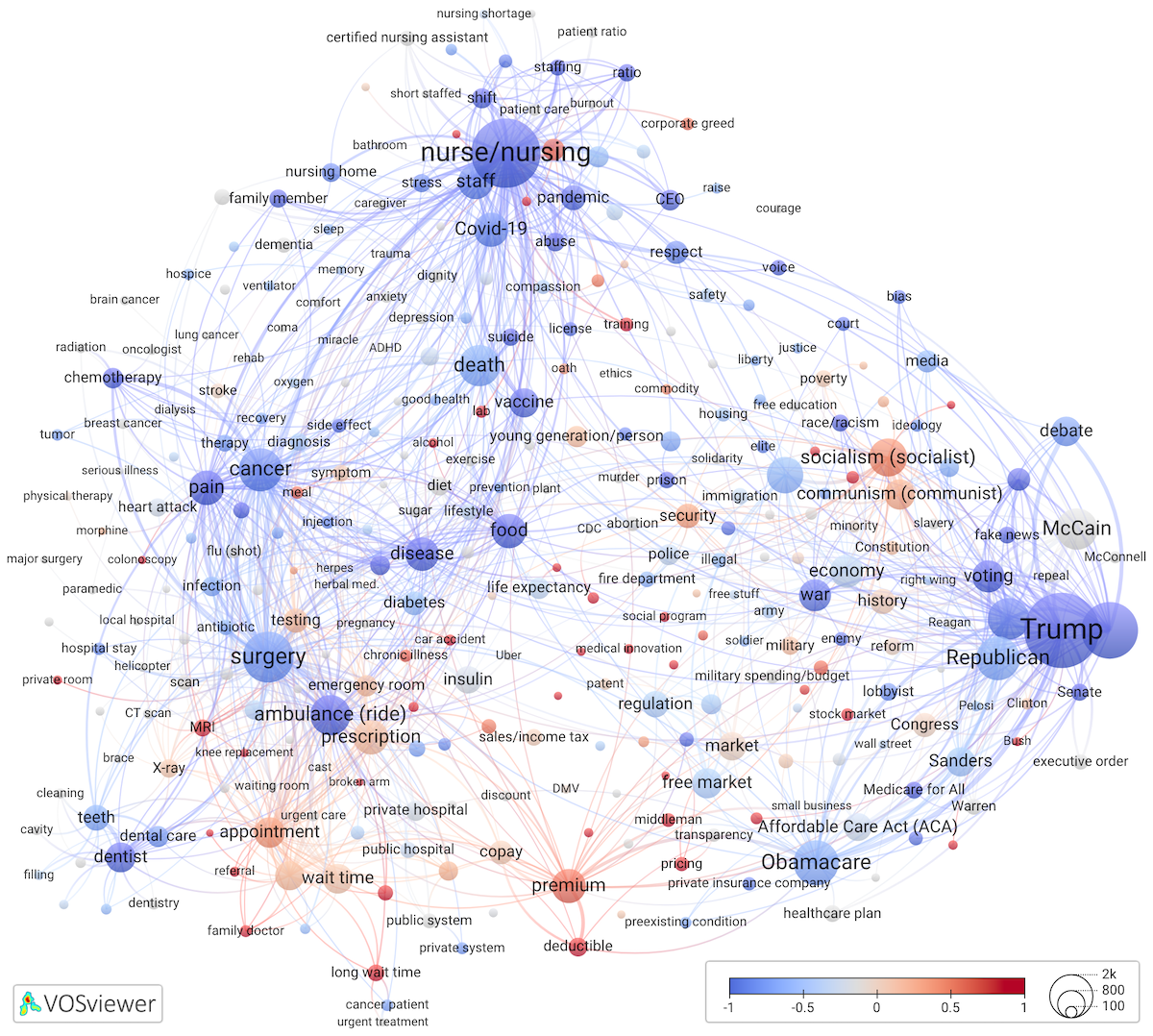


# Supplementary Tables

## Table S1

Video group characteristics: Number of videos per group, degree of left leaning, and YouTube channel owners.

| Video groups | Videos | Degree of Left leaning | | Videos uploaded by: |
| --- | --- | --- | --- | --- |
|  | N | M | SD |  |
| Health care costs and Financial Issues | 13 | 2.46 | 0.52 | CNBC, NYT, PBS, TED, VICE, Vox |
| Health care policies and Politics | 9 | 1.89 | 0.93 | CNN, Fox News, NBC |
| ACA/Obamacare health care Reform | 8 | 2.50 | 0.93 | CNN, Fox News |
| Health care systems in different countries | 8 | 2.38 | 0.52 | CNBC, NowThis, NYT, Washington Post |
| Health care workforce | 7 | 2.43 | 0.79 | ABC News, Amanpour & Company, CBN News, NYT, PBS Vitals, WGN News |
| End-of-life health care | 3 | 3.00 | 0.00 | CNN, PBS |
| Single payer health care | 2 | 2.00 | 1.41 | Fox News, Vox |
| Children’s health care | 1 | 3.00 | n/a | PBS |
| Comedy on the U.S. health care | 1 | 2.00 | n/a | Netflix |
| Medicare for All video by John Oliver | 1 | 3.00 | n/a | LastWeekTonight HBO |

## Table S2

Video group characteristics: Mean video upload year, sum of comments in corpus, total comments, and percentage of total comments in analysis.

| Video groups | Mean video upload year | Comments in corpus, sum | Total comments, sum | Percentage of total comments in analysis |
| --- | --- | --- | --- | --- |
| Health care Costs and Financial Issues | 2019.22 | 45,028 | 68,880 | 66% |
| Health care Policies and Politics | 2020.63 | 21,501 | 30,656 | 74% |
| ACA/Obamacare Health care Reform | 2017.48 | 12,577 | 17,489 | 72% |
| Health care Systems in Different Countries | 2020.09 | 54,691 | 86,401 | 63% |
| Health care Workforce | 2022.35 | 16,748 | 20,832 | 81% |
| End-of-life Health care | 2023.26 | 9,334 | 11,907 | 79% |
| Single Payer Health care | 2014.90 | 1,072 | 1,874 | 57% |
| Children’s Health care | 2022.64 | 1,082 | 1,218 | 89% |
| Comedy on the U.S. Health care | 2022.29 | 763 | 1,089 | 70% |
| Medicare for All video by J. Oliver | 2020.13 | 2,1983 | 40,754 | 54% |

# References

1. Media bias rating method. AllSides. URL: <https://www.allsides.com/media-bias/media-bias-rating-methods> [accessed 2024-03-09]
2. van Eck NJ, Waltman L. VOSviewer manual. Universiteit Leiden. Oct 31, 2023. URL: <https://www.vosviewer.com/documentation/Manual_VOSviewer_1.6.20.pdf> [accessed 2025-01-27]
